# Development of Photopolymerizable Implants for Controlled Release of Pro-Apoptotic 1,2,4-Oxadiazoles

**DOI:** 10.1021/acsomega.4c09142

**Published:** 2025-05-07

**Authors:** Rayane C. G. Aciole, Maria J. S. Lima, Erwelly B. de Oliveira, Dayane K. D. N. Santos, Jaciana S. Aguiar, Severino Alves, Janaína V. dos Anjos

**Affiliations:** † Departamento de Química Fundamental, Universidade Federal de Pernambuco, Recife, Pernambuco 50740-560, Brazil; ‡ Departamento de Antibióticos, Universidade Federal de Pernambuco, Recife, Pernambuco 50740-560, Brazil

## Abstract

We present a study on developing photopolymerizable implants for the controlled release of pro-apoptotic 1,2,4-oxadiazoles to enhance their efficacy and safety in cancer treatment. The research focuses on synthesizing, testing, and incorporating 3,5-diaryl-1,2,4-oxadiazoles into a polymeric matrix based on methacrylates and utilizing these photopolymerizable devices for cancer therapy. Swelling tests showed that while the resin swells in contact with liquids, the presence of oxadiazole slowed this swelling, leading to a prolonged drug release over 50 days. The implant retained the cytotoxic activity of the isolated drug, indicating its potential for cancer therapy.

## Graphical abstract

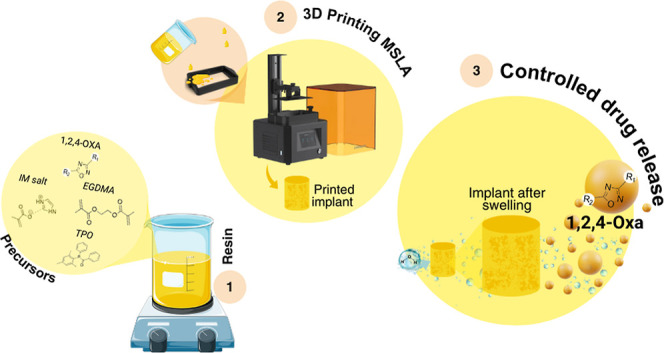

## Introduction

Cancer chemotherapy, a widely used treatment method, involves the use of chemical substances, either alone or in combination, to combat neoplasms that disrupt cell growth and division, leading to the destruction of tumor cells.^1^ However, a significant challenge in antineoplastic chemotherapy is its selectivity in targeting only neoplastic cells. This lack of specificity often leads to the destruction of healthy cells, resulting in severe side effects. This issue underscores the need for more targeted and selective treatment methods.^2^ To address this issue, many researchers have focused on discovering new chemotherapeutic strategies that selectively target malignant cells, thereby enhancing the efficacy and safety of cancer treatment.

The 1,2,4-oxadiazole 3,5-disubstituted compounds, an emerging class of chemotherapeutic agents, hold significant promise in cancer chemotherapy.^3–5^ These substances, known for their antineoplastic properties, induce apoptosis by activating caspases.^6^ These proteolytic enzymes, produced in eukaryotic cells as inactive zymogens, play a crucial role in the selective destruction of neoplastic cells, leading to programmed cell death.^7^

While mechanisms involving apoptosis are preferred in the mechanism of action of an anticancer agent due to better treatment selectivity,^8^ the specific targeting of these substances to the site of action remains a complex challenge.^9^ One potential solution is to incorporate these agents into polymeric matrices designed to release the compounds at specific sites in the body.^10^ This can be achieved using polymeric implants.^11^ These devices can be manufactured using various polymers, and acrylate-based systems are a traditional option in the biomedical field.^12^ They can offer a solution to the challenge of site-specific drug delivery.

With the advent of advanced technology and research, more efficient manufacturing techniques like additive manufacturing or 3D printing have emerged.^13^ These techniques enable the production of polymerizable materials, facilitating fast and precise manufacturing.^14^ This technology opens new avenues for customizing chemotherapy, where treatments can be tailored to individual patients.^15^ This is the essence of personalized medicine (PM). PM, which considers individual variability, lifestyle, and environment, allows for selecting the most suitable clinical pathway for each patient. Given the tumor heterogeneity and individual variability that results in different treatment responses, PM holds the potential to revolutionize cancer treatment by personalizing patient-centered therapies, thereby achieving successful treatment outcomes.^16^

Therefore, the proposal to manufacture implants based on photopolymerizable resins through 3D printing is a challenge that has been gaining traction in this technology, which is still under development and showing promising indications for using these materials in drug delivery. In this regard, the present work aims to present a study on the transport and controlled release of 3,5-diaryl-1,2,4-oxadiazoles using photopolymerizable resins for cancer therapy.

## Experimental Section

### General

Reagents and solvents were purchased from Sigma-Aldrich (USA), Dinâmica (Brazil), and Merck (Germany). No additional purification was required, except for pyridine, which was previously dried with potassium hydroxide and distilled. All reactions were monitored by TLC (thin-layer chromatography) analysis plates containing GF_254_ from Sigma-Aldrich (USA). Compounds were characterized by an Electro-thermal Mel-temp apparatus (Bibby Scientific, UK) to determine melting points. ^1^H and ^13^C nuclear magnetic resonance (NMR) spectra were recorded with a Varian UNMRS 400 MHz (Varian, USA) spectrometer employing deuterated chloroform (CDCl_3_) as solvent. High-resolution mass spectra (HRMS) were recorded on a Bruker Daltonics-micro TOF spectrometer (Bruker, USA). The absorption spectra of the samples were obtained using a Shimadzu UV-2600 spectrophotometer (Shimadzu, Japan) equipped with deuterium and tungsten halogen lamps in the range of 400 to 200 nm. Ethylene glycol dimethacrylate (EGDMA) was synthesized based on prior literature,^17^ and its structure matched the expected (see Supporting Information). Imidazole salt of methacrylic acid was prepared according to de Albuquerque and co-workers.^18^ The Supporting Information file contains data for all synthesized new compounds, including chemical characterization and NMR information. The statistical analysis was performed using Origin 2018 software, and the graphs were plotted using Origin 2018 or Graph Prism 9 software.

### Synthesis of Arylamidoximes **2a–d**

Arylamidoximes were synthesized according to earlier literature.^19^ 0.0970 mol of the corresponding aromatic nitrile (**1a–d**) was dissolved in 100 mL of ethanol in a round-bottom flask. Next, 0.485 mol (33.71 g, 5 equiv) of hydroxylamine hydrochloride (NH_2_OH·HCl) and 0.485 mol (40.74 g, 5 equiv) of sodium bicarbonate (NaHCO_3_) were dissolved in 50 mL of distilled water (each) in separate beakers, followed by the addition of the second solution to the first. This final solution was poured into the round-bottom flask containing the nitrile and stirred for 48 h at room temperature. The progress of the reaction was monitored by TLC (hexane/ethyl acetate, 7:3, v/v) until the nitrile was consumed entirely. The solvent was evaporated, and the residue was extracted using ethyl acetate (3 × 30 mL). The organic phases were combined and dried with anhydrous sodium sulfate. The solvent was evaporated, and the product was obtained by recrystallization in chloroform/hexane. Since the arylamidoximes were used only as a precursor for the subsequent syntheses, and these substances are already well-reported in the literature,^19^ their complete characterizations are not described in this work.

### Synthesis of 3,5-Diaryl-1,2,4-oxadiazoles **4a–l**

1,2,4-Oxadiazoles were synthesized according to the methodology published by dos Anjos and colleagues (2007).^20^ The corresponding arylamidoxime (**2a**–**d**, 7.30 mmol, 1 equiv) was initially placed into a round-bottom flask, and pyridine (10 mL) was added to solubilize the amidoxime. Then, 11.0 mmol (1.5 equiv) of the corresponding acyl chloride **3a**–**c** (used as purchased) was added to the mixture, which was stirred at reflux for 24 h. The reaction progress was monitored by TLC (hexane/ethyl acetate, 7:3, v/v) until the arylamidoxime was consumed. Once the reaction was finished, the mixture was poured into a beaker containing ice, and water and concentrated hydrochloric acid were added until acidic pH was reached, resulting in the formation of a precipitate. This precipitate was filtered and washed with distilled water and a saturated sodium bicarbonate solution to remove excess acid chloride or carboxylic acid. The residue was then recrystallized in a mixture of methanol and water to yield the corresponding oxadiazoles **4a**–**l**. Compounds **4a**,^21^ **4b**,^22^ **4d**,^23^ **4g**,^23^ **4i**,^24^ **4j**,^25^ and **4l** ^26^ are already described in the literature, and their physicochemical properties are according to earlier findings. The five resulting compounds are novel, and their experimental descriptions are presented below.

#### 3-(4-Methoxyphenyl)-5-phenyl-1,2,4-oxadiazole (**4c**)

Yellow solid; yield: 53%; mp 93–94 °C; *R*_f_: 0.73 (7:3, hexane/ethyl acetate, v/v). ^1^H NMR (CDCl_3_, 400 MHz): δ (ppm) 3.79 (s, 3H, CH_3_); 6.92–6.94 (dd, 2H, *J* = 8.8 Hz, 2.0 Hz, Haromatic); 7.43–7.53 (m, 3H, Haromatic); 8.01–8.04 (dd, 2H, *J* = 8.8 Hz, 2.0 Hz, Haromatic); 8.11–8.14 (dd, 2H, *J* = 8.4 Hz, 2.0 Hz, Haromatic). ^13^C NMR (CDCl_3_, 100 MHz): δ (ppm) 55.3; 114.2; 119.4; 124.4; 128.1; 129.0; 129.1; 132.6; 161.9; 168.6; 175.4. HRMS-ES (positive mode): 253.0977 (calcd for C_15_H_12_N_2_O_2_ + H^+^); 253.0972 (found).

#### 3-(4-Chlorophenyl)-5-(furan-2-yl)-1,2,4-oxadiazole (**4e**)

Yellow solid; yield: 47%; mp 110 °C; *R*_f_: 0.80 (7:3, hexane/ethyl acetate, v/v). ^1^H NMR (CDCl_3_, 400 MHz): δ (ppm) 6.58 (dd, 1H, *J* = 3.5 Hz, 1.6 Hz, Hfuran); 7.30 (dd, 1H, *J* = 3.5 Hz, 0.8 Hz, Hfuran); 7.40 (d, 2H, *J* = 8.8 Hz, Haromatic); 7.64 (dd, 1H, *J* = 1.6 Hz, 0.8 Hz, Haromatic); 8.02 (d, 2H, *J* = 8.8 Hz, Haromatic). ^13^C NMR (CDCl_3_, 100 MHz): δ (ppm) 112.5; 116.6; 125.0; 128.9; 129.2; 137.5; 140.0; 146.8; 167.7; 167.9. HRMS-ES (positive mode): 247.0274 (calcd for C_12_H_7_N_2_ClO_2_ + H^+^); 247.0264 (found).

#### 3-(4-Methoxyphenyl)-5-(furan-2-yl)-1,2,4-oxadiazole (**4f**)

White solid; yield: 47%; mp 79–80 °C; *R*_f_ 0.73 (7:3, hexane/ethyl acetate, v/v). ^1^H NMR (CDCl_3_, 400 MHz): δ (ppm) ^1^H NMR (CDCl_3_, 400 MHz): δ (ppm) 3.79 (s, 3H, CH_3_); 6.56 (dd, 1H, *J* = 3.6 Hz, 1.7 Hz, Haromatic); 6.92 (d, 2H, *J* = 8.8 Hz, Haromatic); 7.24 (dd, 1H, *J* = 3.6 Hz, 0.8 Hz, Hfuran); 7.63 (dd, 1H, *J* = 1.7 Hz, 0.8 Hz, Hfuran); 8.02 (d, 2H, *J* = 8.8 Hz, Haromatic). ^13^C NMR (CDCl_3_, 100 MHz): δ (ppm) 55.3; 112.4; 114.2; 116.4; 118.9; 129.2; 140.2; 146.6; 162.0; 167.3; 168.3. HRMS-ES (positive mode): 243.0770 (calcd for C_13_H_10_N_2_O_3_ + H^+^); 243.0769 (found).

#### 3-(4-Chlorophenyl)-5-(thiophen-2-yl)-1,2,4-oxadiazole (**4h**)

White solid; yield 46%; mp 128–129 °C; *R*_f_ 0.87 (7:3, hexane/ethyl acetate, v/v). ^1^H NMR (CDCl_3_, 400 MHz): δ (ppm) 7.14 (dd, 1H, *J* = 5.0 Hz, 4.0 Hz, Hthiophene); 7.39 (d, 2H, *J* = 8.8 Hz, Haromatic); 7.58 (dd, 1H, *J* = 5.0 Hz, 1.2 Hz, Hthiophene); 7.87 (dd, 1H, *J* = 4.0 Hz, 1.2 Hz, Haromatic); 8.00 (d, 2H, *J* = 8.8 Hz, Haromatic). ^13^C NMR (CDCl_3_, 100 MHz): δ (ppm) 125.2; 125.7; 128.5; 128.8; 129.1; 132.0; 132.01; 137.4; 168.0; 171.5. HRMS-ES (positive mode): 263.0046 (calcd for C_12_H_7_N_2_SClO + H^+^); 263.0040 (found).

#### 3-(Thiophen-2-yl)-5-(furan-2-yl)-1,2,4-oxadiazole (**4k**)

White solid; yield 55%; mp 104–105 °C; *R*_f_ 0.84 (7:3, hexane/ethyl acetate, v/v). ^1^H NMR (CDCl_3_, 400 MHz): δ (ppm) ^1^H NMR (CDCl_3_, 400 MHz): δ (ppm) 6.57 (dd, 1H, *J* = 3.6 Hz, 1.8 Hz, Hfuran); 7.09 (dd, 1H, *J* = 5.0 Hz, 3.7 Hz, Hthiophene); 7.30 (dd, 1H, *J* = 3.6 Hz, 0.8 Hz, Hfuran); 7.45 (dd, 1H, *J* = 5.0 Hz, 1.2 Hz, Hthiophene); 7.64 (dd, 1H, *J* = 1.8 Hz, 0.8 Hz, Hfuran); 7.80 (dd, 1H, *J* = 3.7 Hz, 1.2 Hz, Hthiophene). ^13^C NMR (CDCl_3_, 100 MHz): δ (ppm) 112.5; 116.9; 127.9; 129.4; 129.9; 139.9; 146.8; 164.7; 167.4. HRMS-ES (positive mode): 219.0228 (calcd for C_10_H_6_N_2_SO_2_ + H^+^); 219.0176 (found).

### Cytotoxicity Activity

The cytotoxic activity was conducted in vitro through cell growth inhibition assays based on the MTT, 3-(4,5-dimethylthiazol-2-yl)-2,5-diphenyltetrazolium bromide, method using the HeLa (human cervical cancer from Henrietta Lacks), HT-29 (human colon cancer), MCF-7 (human breast cancer), NCIH-292 (human mucoepidermoid lung carcinoma), and VERO (green monkey kidney fibroblasts) cell lines (10^5^ cells/mL) in 96-well plates. The cell lines used in this assay were purchased from the Rio de Janeiro Cell Bank and maintained at the Laboratory of in vitro and in vivo Experimentation Laboratory of the Antibiotic Department at UFPE. The cell lines were tested for mycoplasmas in advance.

The cells were cultured in Roswell Park Memorial Institute medium (RPMI 1640) or Dulbecco’s modified Eagle medium (DMEM), enriched with fetal bovine serum inactivated (10%) and antibiotic (1%), added to 96-well plates, and incubated for 24 h at 37 °C in a 5% CO_2_ atmosphere, after which 10 μL of each one of oxadiazoles **4a**–**l** solutions were added to the wells at a final concentration of 50 μM (at 0.5% DMSO in PBS). Only cell lines with over 95% confluence were plated. A negative control containing only DMSO (dimethyl sulfoxide) and culture medium was added to the cell cultures during the cytotoxicity tests, representing 100% cell viability. The drug doxorubicin was used as a positive control (50 μM). After 72 h of reincubation, 25 μL of MTT (5 mg/mL) was added, and after 3 h of incubation, the culture medium with MTT was aspirated, and 100 μL of DMSO was added to each well for formazan quantification. The absorbance was measured using a microplate reader at a wavelength of 560 nm. The experiments were performed in triplicate.

### Resin Preparation

The preparation of the resin followed this protocol: initially, 2,4,6-trimethyl benzoyl-diphenylphosphine (TPO) (2.72 × 10^–4^ mol; 0.01 equiv; 1 mol %; 0.095 g) was weighed as the photoinitiator and imidazole salt of methacrylic acid (2.72 mol × 10^–2^ mol; 1 equiv, 4.2 g) in a 25 mL round-bottom flask containing a magnetic stirrer. Subsequently, the cross-linker EGDMA (2.72 × 10^–4^ mol; 0.01 equiv; 1 mol %, 0.054 g) was incorporated into the mixture and stirred again. All the procedures were performed in dark conditions to avoid premature polymerization.

For the drug-containing resin, 100 mg of oxadiazole **4e** was added to 4 mL of resin and homogenized using an ultrasonic bath (Branson B2510E-DTH ultrasonic bath operating at 40 kHz, Branson Ultrasonics, USA). This resulted in a resin containing 25 mg of oxadiazole per mL of photocurable resin. This was the maximum amount of oxadiazole **4e** that could be wholly solubilized in the resin.

### 3D Printing

The printing process was performed using the Anycubic Photon Mono 4K printer (Anycubic, China), enhanced by a modified prototype vat characterized by its minimal resin volume requirement (2 mL minimum). We created the modified vat and platform models; the files are available at cults3d.com.^27^ Using the adapted vat and platform also required adding an end-stop extension to prevent the platform from grounding into the LCD screen.^28^ The objects were designed using MeshMixer software and imported into the printer’s software with the output file extension.pwma. Twenty cylinders arranged in four rows with dimensions of 2.5 mm by 2.5 mm and a layer thickness of 0.05 mm were printed during the experiments. The objects were printed using an exposure time of 30 s per layer. In each print, 2 mL of resin containing or not the oxadiazole **4e** was placed in the adapted vat. After each printing cycle, the fabricated pieces were washed using isopropyl alcohol for 1 min, followed by an equal-length curing time. 6.0 × 6.0 mm cylinders were printed using the same printing parameters to measure Schore hardness. For mechanical tests, specimens measuring 30.0 × 4.0 × 2.0 mm specimens, size 1BB, were also printed using the same printing parameters.

### Resin Characterization

Object dimensions were measured using a digital pachymeter (KingTools, Brazil). Shore D hardness (Romacci, Brazil) was performed using a portable digital durometer in 6.0 × 6.0 mm cylinders (ASTM D2240). FTIR was obtained with a diamond crystal (Bruker Alpha-II, USA) in ATR mode. Mechanical tests were conducted using specimens for tension and stress (30.0 × 4.0 × 2.0 mm specimens, size 1BB), following the ISO 527 standards for tensile tests. Each assay was performed in triplicate, and results are expressed by means ± standard deviation.

### Swelling Tests

In this test, specimens loaded with oxadiazole **4e** and unloaded (cylinders measuring 2.5 mm × 2.5 mm) were immersed in a flat-bottom flask containing 5 mL of phosphate-buffered saline (PBS) buffer. The objects were observed, and their masses were measured for 5 days. The increase in mass was calculated based on the measurements of the mass of the cylinder at the time of measurement compared to the measurement on day 0, using the following formula: (mass_final_ – mass_initial_/mass_initial_) × 100%. The swelling studies were conducted at room temperature and 35.7 °C, the average human body temperature. Each assay was performed in triplicate, and results are expressed by means ± standard deviation.

### Preparation of Oxadiazole **4e** Working Solutions

The oxadiazole mother solution was prepared by dissolving 1 mg of oxadiazole **4e** per mL of dimethyl sulfoxide (DMSO). Working solutions were prepared by serial dilutions from the mother solution in phosphate-buffered saline (PBS) buffer. Each solution was transferred to a 10 mL volumetric flask and diluted using PBS buffer. Six different concentrations of oxadiazole **4e** (0.005, 0.00375, 0.00250, 0.00125, 0.00187, 0.000625 mg/mL) diluted in PBS were prepared to estimate absorbance and build a calibration curve. Each assay was performed in triplicate, and results are expressed by means ± standard deviation.

### Determination of Oxadiazole **4e** UV Spectrum

This study used an Agilent Cary 60 UV–vis spectrophotometer (Agilent, USA). After serial dilution with different buffers, the solutions containing various concentrations of compound **4e** were scanned from 200 to 400 nm to select the maximum wavelength (λ_max_). The solutions showed maximum absorption at 280 nm.

### Oxadiazole Release Tests

All materials used in this procedure, such as glassware, printed specimens, and PBS buffer, were sterilized in an autoclave. The drug’s printed specimens were placed into flat-bottom flasks containing 5 mL of PBS and studied for 50 days at room temperature (25 °C) and 35.7 °C using a digital dry bath (Novatecnica, Brazil). The drug release was analyzed continuously over 50 days. At each predefined analysis time, 3 mL of the sample was taken and read in a spectrophotometer to determine the absorbance and calculate the drug concentration released over time. In all analyses, except the first one (0 h), the phosphate-buffered saline (PBS) was periodically replaced into the flask (3 mL) to prevent drug saturation. Each assay was performed in triplicate, and results are expressed by means ± standard deviation.

### Resin Cytocompatibility Test

VERO epithelial cell lines were cultured in RPMI-1640 medium (Sigma-Aldrich, USA) with 10% fetal bovine serum (FBS) (Gibco, Invitrogen, UK) and incubated at 37 °C at 5% CO_2_. The cells were lifted with 0.25% trypsin (Gibco, Invitrogen) for 4 min at 37 °C.

All materials used in this procedure, such as glassware, printed specimens, and PBS buffer, were sterilized in an autoclave. Cell viability was determined using the in vitro Toxicology Assay Kit—MTT Based (Sigma-Aldrich, USA). To perform the assay, 1 × 10^6^ cells were cultivated in a 6-well plate and incubated for 24 h to ensure cell adhesion. After this time, the 2.5 by 2.5 mm cylindric resin specimen was added to the well—the negative control contained only cells and culture medium. After 24 h of incubation, MTT (3-[4,5-dimethylthiazol-2-yl]-2,5-diphenyltetrazolium bromide, 100 μL from solution at 3 mg/mL in PBS) was added to each well, and the plates were incubated at 37 °C in a humid atmosphere containing 95% air and 5% CO_2_ for 3 h. Finally, 1 mL of solubilization solution is used to solubilize formazan crystals. Absorbance was measured using a plate reader (Multiskan SkyHigh, Thermo Fisher Scientific, USA) at 560 nm. The experiments were performed in triplicate.

### Microscopic Images

Images from the printed pieces were assessed on a USB (Universal Serial Bus) digital microscope, Play 16 (PlayShop, Brazil).

### Scanning Electron Microscopy (SEM)

Cylindrical specimens loaded with oxadiazole **4e** were coated with gold and visualized in the Tescan Mira 3 apparatus (Tescan, Czech Republic) with an acceleration voltage between 5 and 10 kV to assess the surface morphology before and after swelling tests.

### Oxadiazole-Loaded Resin Cytotoxicity Test in NCIH-292 Cell Line

Mucoepidermoid lung carcinoma cells (NCIH-292) were cultured in RPMI-1640 medium (Sigma-Aldrich, USA) with 10% fetal bovine serum (FBS) (Gibco, Invitrogen, UK) and incubated at 37 °C at 5% CO_2_. The cells were lifted with 0.25% trypsin (Gibco, Invitrogen) for 4 min at 37 °C.

All materials used in this procedure, such as glassware, printed specimens, and PBS buffer, were sterilized in an autoclave. Cell viability was determined using the in vitro Toxicology Assay Kit—MTT Based (Sigma-Aldrich, USA). To perform the assay, 1 × 10^6^ cells were cultivated in a 6-well plate and incubated for 24 h to ensure cell adhesion. After this time, the 2.5 by 2.5 mm cylindric resin specimen containing oxadiazole **4e** was added to the well—the negative control contained only cells and culture medium. After 24 h of incubation, 100 μL of MTT solution at 3 mg/mL in PBS was added to each well, and the plates were incubated at 37 °C in a humid atmosphere containing 95% air and 5% CO_2_ for 3 h. Finally, 1 mL of solubilization solution is used to solubilize formazan crystals. Absorbance was measured using a plate reader (Multiskan SkyHigh, Thermo Fisher Scientific, USA) at 560 nm. The experiments were performed in triplicate.

### Immediate Release Assay of Oxadiazole **4e** from the Printed Matrix

A unit of the printed specimen containing oxadiazole **4e**, measuring 2.5 × 2.5 mm, was macerated using a mortar and pestle until the sample was completely pulverized. Then, 10 mL of ethanol was added to this powder and subjected to ultrasonic treatment (Branson B2510E-DTH ultrasonic bath operating at 40 kHz, Branson Ultrasonics, USA) for 30 min to extract the oxadiazole. After this period, 1 mL of this ethanolic solution was diluted with more ethanol to form a 30 mL solution. This diluted solution was homogenized and analyzed using an Agilent Cary 60 UV–vis spectrophotometer (Agilent, USA) at 257 nm (maximum absorption using ethanol). The concentration of oxadiazole **4e** was measured using a calibration curve of **4e** dissolved in ethanol (concentrations ranging from 4.5 × 10^–5^ to 1.5 × 10^–3^ mg/mL; curve available in the Supporting Information). In this case, the total drug concentration after dilutions should be 9.7 × 10^–4^ mg/mL if the release is 100%. This assay was carried out in triplicate.

## Results and Discussion

The 3,5-diaryl-1,2,4-oxadiazole series was synthesized using the classical methodology involving the cyclization of arylamidoximes to reactive carboxylic derivatives.^20^ The arylamidoximes (**2a**–**d**) were synthesized from the corresponding aromatic nitriles (**1a**–**d**) by reaction with hydroxylamine in a hydroethanolic medium. Once isolated and purified, the arylamidoximes reacted with the respective acyl chlorides (**3a**–**c**) in pyridine under reflux to furnish the corresponding oxadiazoles **4a**–**l** in moderate chemical yields (Scheme 1).

**Scheme 1:**
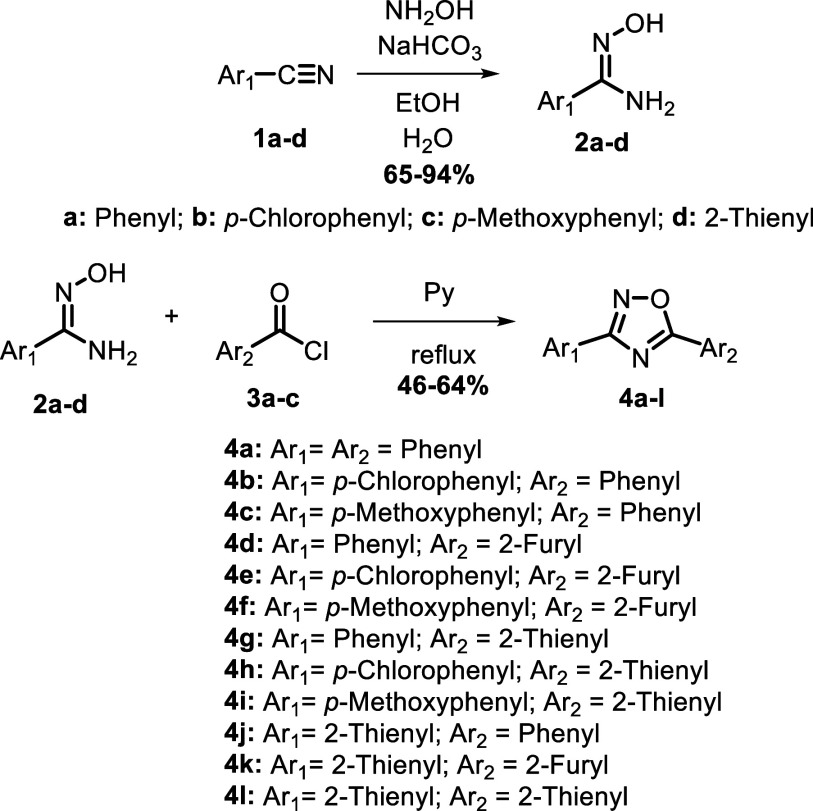
Synthesis of 3,5-Diaryl-1,2,4-oxadiazoles **4a**–**l**

These tricyclic systems have already been described as cytotoxic agents in cancer cells by activating apoptosis. Jessen and co-workers conducted studies on the biological target of cytotoxic 3,5-diaryl-1,2,4-oxadiazoles.^29^ These researchers discovered that the molecular target of these substances is a carrier protein, TIP47 (tail interacting protein of 47 kDa). This cytosolic protein facilitates the transport of mannose 6-phosphate receptors (or IGF-2, insulin-like growth factor-2) from the endosomes to the Golgi complex, which is crucial for recycling these receptors. Thus, if there is insufficient TIP47 for recycling these receptors (IGF-2R, M6PR), the complexes proceed to the lysosomes and are digested.^30^

Jessen and collaborators also found an increase in the expression of TGF-β1 (tumor growth factor-beta 1), IGF-BP3 (insulin-like growth factor-binding protein-3), and p21 genes, along with a decrease in the expression of the cyclin D1-related gene.^29^ These gene alterations lead to apoptosis in eukaryotic cells. Based on these findings, it can be inferred that these molecules’ mechanism of action involves binding to TIP47, which is related to apoptosis.

Inspired by these results, we tested this series of 12 3,5-diaryl-1,2,4-oxadiazoles on different cancer cell lines and VERO cells to evaluate cytotoxicity. These results are compiled in Table 1.

**Table 1:** Cytotoxic Activity (%) of Oxadiazoles **4a**–**l** on Different Cell Lines at 50 μM^a^

compound	HeLa (cervix cancer)	MCF-7 (breast cancer)	HT-29 (colon cancer)	NCIH-292 (lung carcinoma)	VERO (fibroblast)
**4a**	37.37 ± 1.98	43.39 ± 2.22	11.42 ± 1.59	52.29 ± 2.53	38.90 ± 2.14
**4b**	48.24 ± 1.22	36.63 ± 3.30	18.25 ± 0.47	44.17 ± 0.53	29.31 ± 1.94
**4c**	49.88 ± 3.43	22.98 ± 1.53	39.89 ± 0.93	66,17 ± 0.63	45.01 ± 3.38
**4d**	32.94 ± 2.79	57.93 ± 1.70	27.38 ± 0.39	58.20 ± 0.81	20.67 ± 1.75
**4e**	**75.68** **±** **4.65**	**81.99** **±** **3.19**	**69.93** **±** **1.14**	**83.82** **±** **1.82**	**40.71** **±** **3.14**
**4f**	42.92 ± 1.24	31.77 ± 0.57	50.76 ± 0.62	64.07 ± 3.42	22.97 ± 2.24
**4g**	22.84 ± 2.06	27.78 ± 2.98	23.54 ± 1.08	55.15 ± 4.09	35.28 ± 1.60
**4h**	36.92 ± 0.44	50.18 ± 2.50	56.47 ± 3.95	64.55 ± 4,52	38.81 ± 1.87
**4i**	42.40 ± 3.08	38.57 ± 2.98	50.06 ± 3.67	29.51 ± 1.14	34.06 ± 1.76
**4j**	28.16 ± 1.50	39.20 ± 3.12	16.32 ± 0.46	31.15 ± 6.28	36.24 ± 2.83
**4k**	22.63 ± 2.64	39.36 ± 1.45	23.29 ± 2.04	43.67 ± 6.49	26.42 ± 0.81
**4l**	25.48 ± 0.99	35.74 ± 3.45	27.22 ± 1.87	32.58 ± 3.45	48.36 ± 1.50
doxorubicin	97.02 ± 0.22	67.45 ± 0.70	72.00 ± 1.53	32.66 ± 0.87	66.17 ± 3.45

a Each point represents the mean ± SEM of a triplicate.

After analyzing the results, the most promising compound tested is oxadiazole **4e**, substituted with a *p*-chlorophenyl group at C-3 and a furyl group at C-5. This oxadiazole inhibited the growth of the three cell lines by more than 50%, outperforming doxorubicin in MCF-7 (breast cancer) and NCIH-292 (lung epidermoid cancer) cells. Structurally comparing this oxadiazole with its bioisosteres at C-5 (**4b** and **4h**), it is evident that the replacement by a phenyl ring is detrimental, while the replacement by a thiophene ring provided a derivative with moderate to good activity, but nothing comparable to the performance of **4e**.

The IC_50_ (half-maximal inhibitory concentration) value for **4e** was calculated for the cell growth inhibition in HeLa, MCF-7, and NCIH-292 cell lines, in which inhibition was greater than 75% at 50 μM. These values were determined to be 29.11 ± 2.48 μM, 33.33 ± 3.70 μM, and 22.99 ± 1.68 μM for these cell lines, respectively.

Previous studies have reported that the C-3 position of oxadiazoles should be substituted with an aromatic group containing a chlorine atom at the C-4 position of the aromatic ring and a thienyl group at C-5 for a good anticancer activity.^6,29^ Jessen and co-workers obtained excellent results with their lead compound, MX-126374, 3-(5-chloropyridin-2-yl)-5-(3-chlorothiophen-2-yl)-1,2,4-oxadiazole.^29^ In this study, it was confirmed that TIP-47 was the molecular target of the lead compound. Given the structural similarity, we believe that the target of **4e** should be the same as for 3-(5-chloropyridin-2-yl)-5-(3-chlorothiophen-2-yl)-1,2,4-oxadiazole (Figure 1), leading us to think that the cytotoxic mechanism of action for **4e** is through the activation of caspases and induction of apoptosis.

**Figure 1:**
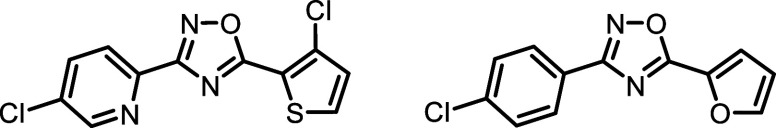
Structures of MX-126374 (left) and **4e** (right).

No binding studies (in silico or in vitro) confirm which regions of TIP47 the 3,5-diaryl-1,2,4-oxadiazoles bind to. However, one study identified the residues important for the binding of TIP47 to the Rab9 protein, which is important for transporting MPRs from late endosomes to the trans-Golgi network. These residues are 161–169 (Gly-Val-Asp-Lys-Tyr-Lys-Ser-Val-Val).^31^ However, it remains unclear whether oxadiazoles would interact with the same region. Therefore, we cannot precisely determine how these structural modifications would affect TIP47 binding affinity.

According to Tsygankova and Zhenodarova,^32^ in a publication where they describe the use of fragment descriptors and linear models obtained by the regression method to accurately calculate the activity of apoptosis inducers, the presence of chlorine atoms in the structure contributes favorably to apoptosis-inducing activity. A descriptor representing the number of pairs of chlorine and aromatic carbon atoms separated by seven chemical bonds indicated that the presence of chlorine is very important for activity. In this article, the authors also report that the absolute value of the contribution of the chlorine descriptor is greater than that of the bromine descriptor. This suggests that substituting the chlorine atom on the aromatic ring in the side chain of the 1,2,4-oxadiazole ring is more favorable than the bromine atom for enhancing the activity of the compounds.

Although compound **4e** was quite active against cancer cells, it was also somewhat active against VERO cells. It had a small cytotoxic potential for noncancer cells,^33^ as cell viability was just under 60% in the presence of **4e**. This indicates that even though there may be an assumed preference for cancer cells, the drug is not highly selective for this type of cell and may also act on noncancer cells.

One way to circumvent this problem is to make use of drug delivery systems. Many systems have already been developed for this purpose, and systems based on biocompatible polymer networks can be highly beneficial in delivering cytotoxic drugs. According to Nayak and colleagues, polymer-based drug delivery systems for chemotherapeutic agents provide several benefits, including stable drug-plasma levels, lower doses with maintained efficacy, reduced dosing frequency, improved bioavailability, minimized side effects, and better patient adherence.^34^

The new drug delivery system developed to serve as a carrier for oxadiazole **4e** has as its structural basis a polymer formed by networks of imidazole salt of methacrylic acid cross-linked with 1% ethylene glycol dimethacrylate (EGDMA) in molar proportion. Higher proportions of cross-linkers were attempted, resulting in nonhydratable or nonswellable objects. Resins prepared with proportions lower than 1% wholly dissolved within 24 h of introducing oxadiazole. Imidazole salt of methacrylic acid resins without cross-linker provided materials completely dissolved within one and a half hours of immersion.^18^

The 6.0 by 6.0 mm cylinders were printed with and without oxadiazole **4e**, and their Shore D hardness was measured on the bench after printing. The prints were successful, and 4 cylinders could be obtained per print. The Shore hardness of the cylinders postprinting without the drug was 65.80 ± 2.66 D, and the hardness of the cylinders containing the drug was 59.50 ± 0.500 D. These values categorize the printed material unloaded with **4e** as hard (between 61 and 80) on the Shore D hardness scale. This characteristic confers greater durability, lifespan, and resistance to wear and impact.^35^ Even after loading the drug, there were no significant changes in hardness. This slight decrease was expected due to the large amount of material introduced into the resin (ca. 0.29 mg per 2.5 × 2.5 mm implant). As the material deposits between the layers of the printed material, it alters intermolecular interactions within the polymeric chains, making the material slightly more fragile.

The printed materials, unloaded and loaded with **4e**, were tested using standard specimens for tensile stress analysis. Specimens were printed along the “*x*” axis to ensure layer orientation perpendicular to fracture stress, enhancing failure resistance. This methodology is essential to the testing process.^36^ Unloaded resin showed excellent mechanical properties, exhibiting an excellent elasticity modulus value of 813 MPa, as seen in Figure 2. This parameter relates the stress required to deform a material longitudinally to the elongation caused by the stress, providing a measure of the stiffness of the printed material. In other words, the stiffer the material, the higher the Young’s modulus (or elasticity modulus).^37^ For the printed material containing the drug, the value of the modulus of elasticity drops significantly, averaging 213.5 MPa (see Supporting Information for more details). This indicates that the drug alters the interactions within the polymer network, resulting in a printed material with lower stiffness. This decrease in value due to the introduction of the drug does not seem to be an issue, as the material will absorb liquids when in contact with bodily fluids in the form of an implant. In the way the measurement was performed, with the material printed and dry, Young’s modulus value for the loaded resin is similar to the maximum value associated with human skin, which is 140 MPa.^38^

**Figure 2:**
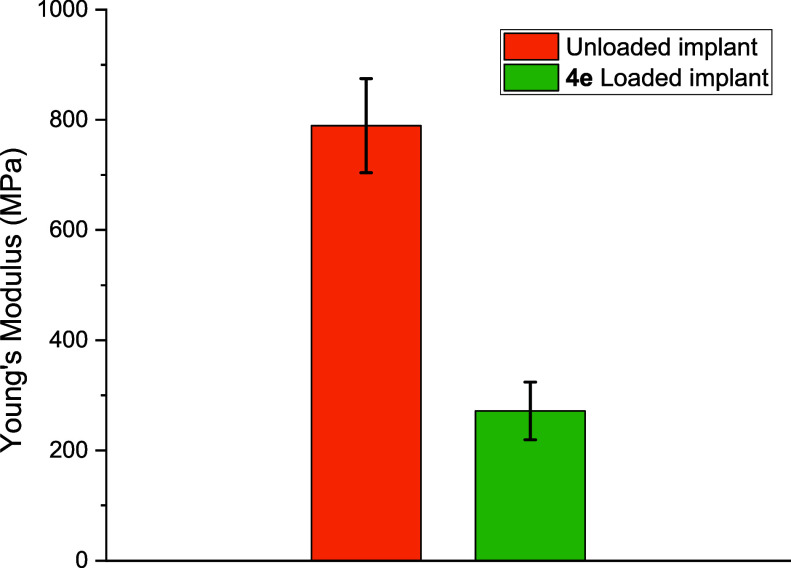
Young Modulus for unloaded printed resin and loaded with oxadiazole **4e**.

The FTIR spectra of the resin loaded with **4e** showed a significant difference compared to those of its precursors, imidazole, methacrylic acid, EGDMA, and **4e**, confirming the formation of this material (Figure 3).

**Figure 3:**
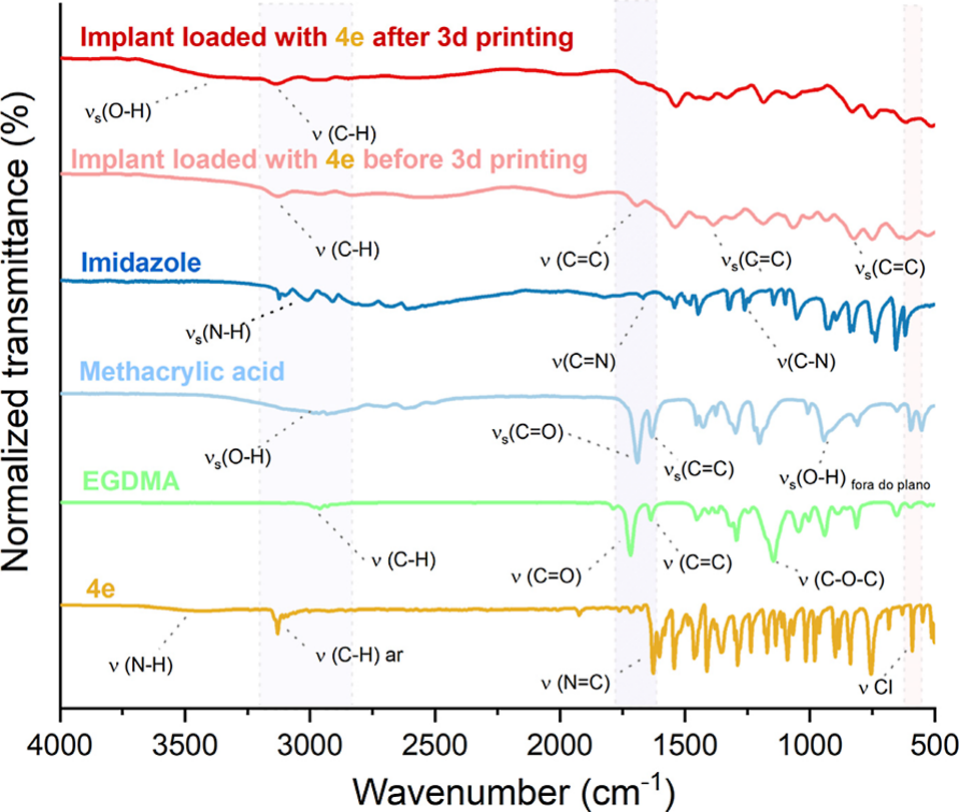
FTIR spectra of the cured resin (red line) compared with its precursors and the uncured resin (pink line): imidazole­(dark blue line); methacrylic acid (light blue line); EGDMA (green line); oxadiazole (yellow line).

The FTIR spectra of the resin loaded with **4e** exhibited a significant difference compared to those of its precursors—imidazole, methacrylic acid, EGDMA, and **4e**—confirming the photopolymerization (Figure 3). In the spectrum of methacrylic acid (light blue line), a broad and intense band was observed between 3100 and 2800 cm^–1^, corresponding to the O–H vibrations present in the structure. At 1700 cm^–1^, a strong band was identified, related to the C=O stretching vibrations of the carboxylic acid, and at 1634 cm^–1^, a signal corresponding to the C=C stretching vibrations due to the vinyl unsaturation in methacrylic acid was detected.^39^

In the spectrum of imidazole (dark blue line), the band corresponding to the N–H vibrations of free imidazole was observed at 3120 cm^–1^, as expected. The band at approximately 2800 cm^–1^ corresponds to the symmetric C–H stretching vibrations. Additionally, bands related to C=N and C–N stretching vibrations were observed at 1675 cm^–1^ and 1250 cm^–1^, respectively.^40^

In the spectrum of EGDMA (light green line), an absorption band at 2959 cm^–1^ can be attributed to the C–H vibrations present in this monomer. The intense band observed at 1716 cm^–1^ corresponds to the C=O vibration of the ester group in EGDMA, confirming that the methacrylate group is attached to the polymer chain terminus via an ester function. A medium-intensity signal was also observed at 1637 cm^–1^, corresponding to the C=C vibration.^41^

In the absorption spectrum of 3-(4-chlorophenyl)-5-(furan-2-yl)-1,2,4-oxadiazole (**4e**) (yellow line), the presence of a slightly broad peak can be attributed to the N–H stretching vibrations in the oxadiazole ring, within the 3500–3300 cm^–1^ region. The strong and broad peak at 3129 cm^–1^ may be related to the C–H stretching vibrations of aromatic bonds. Between 1600 and 1500 cm^–1^, characteristic peaks of C=C stretching vibrations in the aromatic rings of the furan and chlorophenyl groups were observed. At 1627 cm^–1^, a characteristic peak of the N=C bond in the oxadiazole ring was detected. The signals corresponding to the vibrational modes of the furan ring appear in the 1500–1000 cm^–1^ range, and a peak at approximately 600 cm^–1^ can be attributed to the chlorine present in the structure.^41^

It is important to highlight the changes in the spectra of the resins before and after printing (cured; pink and red lines, respectively), demonstrating the successful polymerization of the resin containing **4e**. The signals corresponding to the C=C bonds of the methacrylate group, at 1681, 1388, and 818 cm^–1^, exhibited significantly reduced intensities after printing, confirming the formation of the polymer network as the double bonds were consumed.^42^

The unloaded printed objects were tested for cell viability in the VERO cell line (ATCC CCL-81) using the MTT method.^43^ The result is shown in Figure 4, in which the percentage of cell viability for VERO cells after treatment with the resin was 96.4% compared to the control (100% cell viability). This indicates that the resin exhibits excellent biocompatibility, paving the way for developing pro-apoptotic drug-delivery devices with oxadiazole **4e**.

**Figure 4:**
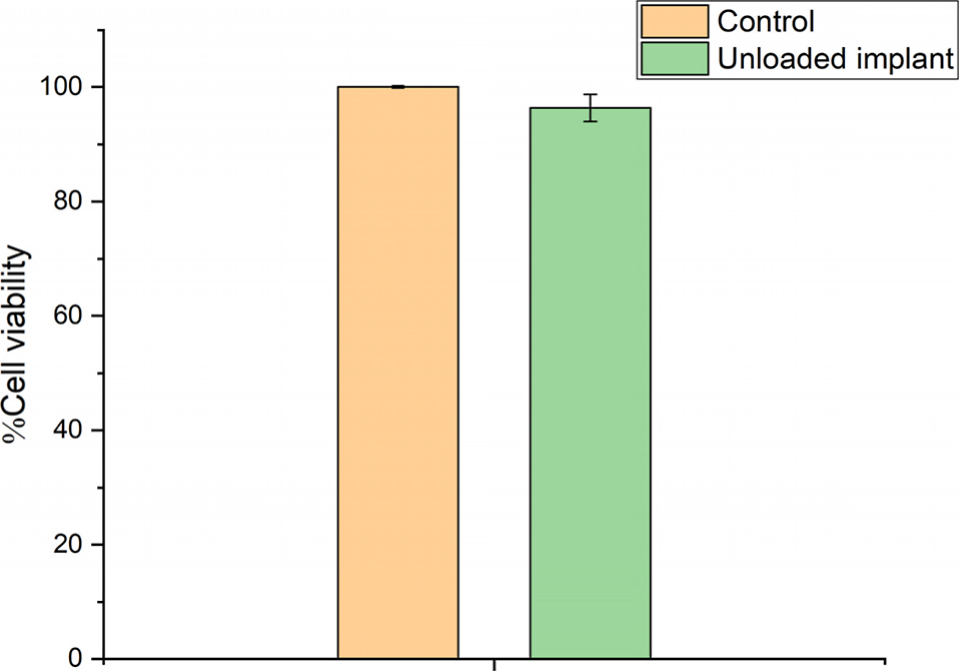
Viability (%) of the VERO cells after 24 h of exposure to resin samples unloaded with **4e**.

Once the resin proved biocompatible, swelling tests and the release of oxadiazole **4e** could be carried out. It was observed that, although they are hard materials at the end of printing when subjected to swelling tests, the cylinders increased significantly in size without suffering dissolution. In the swelling tests using PBS buffer at 25.0 °C with the unloaded resin, the network increased its mass by almost 2500% after 3 days of testing (Figure 5a), reaching its maximum swelling. After this point, the material begins to lose liquid to the surrounding medium, stabilizing at around 1700% of its original size. The swelling is less pronounced with the drug, reaching about 470% after 5 days of testing. It can also be observed that swelling primarily occurs within the first 24 h, with minimal changes in the following days.

**Figure 5:**
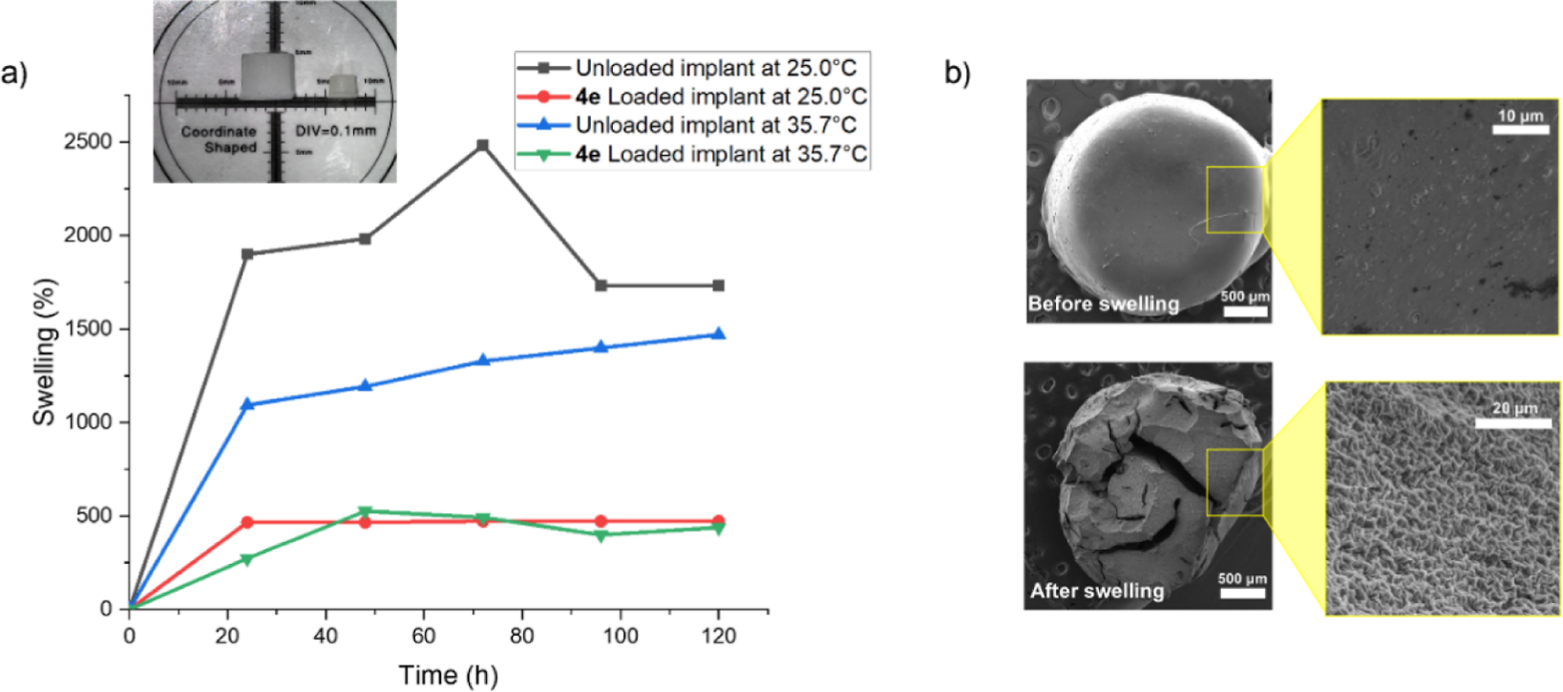
(a) Swelling test for printed implant unloaded (black) and loaded with **4e** (red) at 25.0 °C; unloaded (blue) and loaded with **4e** (green) at 35.7 °C. The inset figure contains the USB microscopic image of the **4e** loaded implant after (left) and before (right) the swelling tests; (b) MEV images of the **4e** loaded implant before (top) and after (bottom) the swelling tests.

In addition to swelling tests at 25.0 °C, swelling tests were also conducted at 35.7 °C, the average human body temperature. In these tests, the object increased its mass by 1500% compared to the original volume after 5 days of testing. Regarding the **4e** loaded material, as observed at 25.0 °C, the swelling was slightly lower, reaching about 400% in the same time frame, as shown in the inset in Figure 5a. This was expected, as introducing oxadiazole **4e**, an aromatic and hydrophobic molecule, would somewhat hinder water entry into the material. Nevertheless, even with the introduction of the drug, the material still exhibited swelling. This characteristic makes this material an ideal substrate for drug delivery through diffusion in a biological medium since it can provide spatial control over the release^44^ of oxadiazole **4e**.

The microstructure of the implants was observed using scanning electron microscopy (SEM). In Figure 5b, SEM images of the implant containing oxadiazole **4e** can be seen before swelling (top) and after swelling (bottom). Noticeably, after swelling, the implant shows a homogeneous and interconnected porous structure, indicating structural stability and a uniform chemical structure. The material’s porosity is essential for the drug retained within it to be released during diffusion.^45^

The material’s porosity is highly relevant in drug delivery systems.^46^ The homogeneous porous structure observed in the implant after swelling suggests an organized network crucial for the efficient diffusion of liquids and, consequently, for drug release.^47^ Porous materials provide channels through which the solvent can penetrate, facilitating the controlled release of the retained drug.^48^ In this case, the porosity allows oxadiazole **4e** to be gradually released, a desirable characteristic for delivery systems. Porosity also affects the network’s structural stability.^49^ Even after swelling, maintaining an interconnected porous network suggests that the material can absorb liquids without compromising its mechanical integrity, showing good stability, which can be advantageous for long-term treatments such as cancer treatments.

As the temperature of the medium increases, the material swells less, both in the resin without the drug and in the resin with the drug, with the most significant difference observed in the unloaded material. This indicates that the material, particularly when not loaded with the drug, is a temperature-sensitive polymer network. According to the literature, the thermal behavior of temperature-sensitive of matrices directly results from the thermosensitivity of the molecular interactions between the polymer network inside the polymer and the solutions within and around the polymer.^50^ For example, in a network made of poly­(*N*-isopropylacrylamide), the efficiency of hydrogen bond formation between the polymer and water molecules decreases at higher temperatures. At lower temperatures (around 22.0 °C), water molecules can efficiently form hydrogen bonds with the amide group in the polymer side chains. As a result of this efficient hydrogen bonding, water molecules move into the polymer network, causing the material to swell. When the temperature increases to 42.0 °C, the efficiency of hydrogen bonding decreases. This process reduces the interaction energy of the polymer network with water molecules, causing water to leave the polymer and shrinking the material.^51^ An increase in temperature generally reverses the osmotic pressure difference, and water exits the matrix, leading to shrinkage. Conversely, a decrease in temperature increases the osmotic pressure difference, causing water to enter the polymer network and resulting in swelling.^50^

Our material is not a poly­(*N*-isopropylacrylamide) network but likely exhibits similar swelling characteristics. Our polymer, primarily made of polymethacrylate of imidazolium as the principal polymer, efficiently forms hydrogen bonds with water in the surrounding medium due to its structural nature. Similar results have been observed with gels containing vinyl-imidazole, with a negative temperature response regarding swelling. The authors believe that at lower temperatures, the hydrophilic nature of the material allows the polymer chains to form intermolecular hydrogen bonds with the surrounding water, leading to swelling. As the temperature increases, these bonds shift to intramolecular hydrogen bonds, causing the material to become more hydrophobic and deswelling.^52^

Before conducting oxadiazole release tests, a calibration curve needed to be constructed. For this purpose, the DMSO stock solution was diluted with PBS buffer to create a series of standard concentrations of oxadiazole **4e** solution ranging from 0.00075 to 0.0050 mg/mL. Absorbances were measured, and by plotting absorbance versus concentration, the calibration curve was constructed, and the regression equation was determined. As shown in the inset in Figure 6, the linear equation was *y* = 208.20 + 0.018, with a correlation coefficient (*R* ^2^) of 0.9943, indicating good linearity.

**Figure 6:**
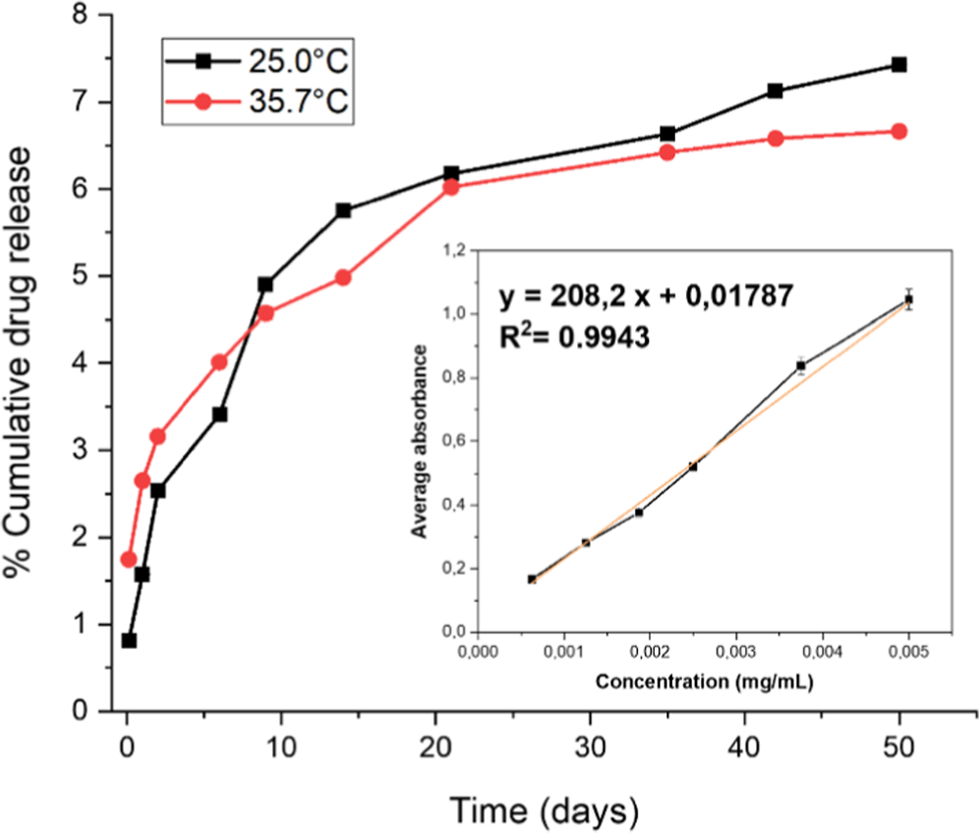
Cumulative oxadiazole **4e** release from photoprinted implant over 50 days at 25.0 and 35.7 °C. The inset figure is the calibration curve for oxadiazole **4e** in DMSO/PBS buffer.

Oxadiazole release tests were conducted after incorporating oxadiazole into resin and printing cylinders measuring 2.5 × 2.5 mm. The aim was to evaluate the drug concentration released over time. The release profile is presented in Figure 6. The oxadiazole **4e** was incorporated into the resin at a concentration of 25 mg of drug per mL of resin. This was the maximum amount of oxadiazole **4e** that could be solubilized in the resin. This way, each 2.5 × 2.5 mm implant (0.0120 mL of average volume) is estimated to contain 0.29 mg of oxadiazole **4e**. At this concentration, the release profile of the oxadiazole was studied over 50 days at room temperature (25.0 °C) and 35.7 °C. By the end of this period, less than 10% of the total was released, highlighting the potential for prolonged effects of the implant in both studied temperatures. This indicates that the system is suitable for applications requiring sustained and long-term release, such as cancer treatments, which is the case. The release is gradual up to around the thirtieth day, after which the release kinetics plateau, showing that the system maintains constant drug levels without significant peaks. No significant differences in drug release are observed at a temperature of 35.7 °C, showing that temperature does not appear to influence the release kinetics in these systems. This way, according to the release assays, an average of 2 μg/mL of the drug was released each day at the beginning of the test. This amount gradually decreased over time, reaching an average of 0.0600 μg/mL per day in the final days of the 50 day test.

According to Son and colleagues,^53^ drug release mechanisms can follow four pathways: diffusion-controlled release, solvent-controlled release, polymer degradation-controlled release, or pH-mediated release. During the swelling and release tests, no polymer degradation was observed, nor was the material subjected to changes in the pH of the medium. Since the drug is uniformly dispersed in the material, the first mechanism does not appear to govern the release. Therefore, it is believed that the release of **4e** is being controlled by the entry of solvent into the material, that is, by the swelling of the polymer network.

The swelling rate of the polymer matrix without the drug is high, but when the drug was introduced into the material, this rate decreased significantly. In other words, introducing oxadiazole **4e** may have affected the drug release from the matrix, making it denser and preventing a more significant amount of liquid from entering, thereby limiting drug release. Although the release was sustained over time, which is good, such a small amount of material was not expected to be released over time.

Previous studies have shown that, in controlled release systems, the swelling rate of the polymer is directly proportional to drug release.^54^ The more the material swells, the more rapidly the drug releases. In dense and highly cross-linked polymers, solvent entry is hindered, resulting in a lower drug release rate.^54,55^

Former results from our research group with these methacrylate systems as cross-linked implants with resorcinol dimethacrylate support these findings. The system was cross-linked with 40 mol % of resorcinol dimethacrylate, using imidazolium methacrylate salt as the main chain. In this case, the implants contained dexamethasone disodium phosphate, a corticosteroid. A lower swelling rate was observed, around 300%, which was expected since the material is more cross-linked. However, it was noted that the drug was fully released in less than 72 h.^56^ Once the drug is more soluble in water and PBS than oxadiazole **4e**, the release profile differs. In other words, the degree of cross-linking may affect the release, but the chemical nature of the drug used plays a central role in the sustained release process. Since oxadiazoles are tricyclic systems with aromatic character, their affinity for (and consequently, solubility in) water or buffer is lower, directly impacting the release rate.

Although the cross-linking agent was used in only 1 mol %, cross-linking can still occur in its absence due to intermolecular and intramolecular chain transfer reactions during free-radical polymerization. In the case of intermolecular reactions, a growing polymer chain can transfer its radical to another polymer chain, leading to the formation of covalent bonds between different chains, and increasing cross-linking within the polymer network. In intramolecular reactions, radical sites within the same chain can react with nearby segments, forming structures that contribute to a denser and more cross-linked network.^57^ These effects are more pronounced when polymerization occurs at high monomer concentrations or when polymer chains contain functional groups capable of forming secondary interactions (such as hydrogen bonding), promoting physical cross-linking.^58^

Another hypothesis (raised by one of the referees who reviewed this work) is that oxadiazole **4e** may have photopolymerized during the printing of the implants. The tricyclic system present in **4e** is relatively stable, as it is fully conjugated, and both the furan ring and the benzene ring substituted with a chlorine atom are considered aromatic. The 1,2,4-oxadiazole ring also has an aromatic character but is better described as a conjugated system.^59^ Among these molecular fragments, the furan moiety is the most likely to react with the matrix. There are reports of radical formation in systems containing the furan ring. For example, Gandini and Rieumont conducted experiments aiming to radicalize furan derivatives in the presence of azo-bis-isobutyronitrile (AIBN) at 78 °C.^60^ In this case, no polymerization products between the furan rings were observed, and the reasons for this failure may be related to the stability of the radical formed in the initial reaction. However, it has been reported that the furan ring can react with acrylates via a Diels–Alder reaction in an acidic medium.^61^ Assuming this could have occurred during vat photopolymerization, **4e** would become covalently bound to the polymer, preventing its release during the release assays.

To investigate this possibility, a 2.5 × 2.5 mm specimen was macerated until fully pulverized and then immersed in an ethanol solution, followed by ultrasonic treatment for 30 min to ensure complete extraction of oxadiazole **4e** from the sample. After spectrophotometric analysis using a new calibration curve in ethanol (available in the Supporting Information), it was determined that the printed specimen contained approximately 51% (51.1 ± 0.809%) of the total amount estimated per implant. Two premises can be considered: first, that a portion of the total **4e** may have indeed reacted with the implant matrix, or second, that the extraction method was not fully effective in recovering all the drug from the sample. Experiments using oxadiazole **4e** were conducted by placing the implant in contact with NCIH-292 cell cultures (Figure 7). In this experiment, the implant containing oxadiazole **4e** inhibited the growth of NCIH-292 cells by 69.90%, as the cell viability was 30.10% compared to the negative control group (no treatment). This shows that our implant containing oxadiazole **4e** can limit tumor cell growth, demonstrating the potential for personalized cancer therapy.

**Figure 7:**
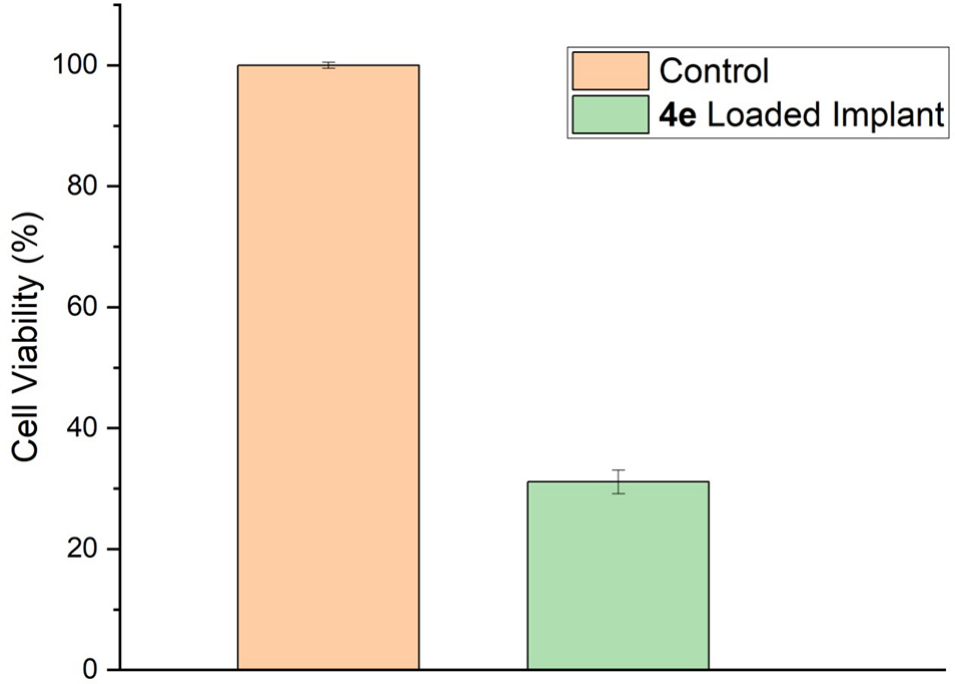
Cell viability on NCIH-292 (lung carcinoma cells).

## Conclusion

Twelve 3,5-diaryl-1,2,4-oxadiazoles with potential anticancer activity were synthesized from arylamidoximes and acid chlorides in moderate to good yields. Of these, 5 are novel. All compounds were tested for cytotoxic activities in 4 cancer cell lines: HeLa (human cervical cancer), HT-29 (human colon cancer), MCF-7 (human breast cancer), NCIH-292 (human mucoepidermoid lung carcinoma), and VERO cells. Among all the tested compounds, 3-(4-chlorophenyl)-5-(furan-2-yl)-1,2,4-oxadiazole (**4e**) stood out, being active in all cancer cell lines.

To create an implant for use in the treatment of neoplasms, oxadiazole **4e** was incorporated into a photopolymerizable resin composed of imidazole salt of methacrylic acid cross-linked with ethylene glycol dimethacrylate (EGDMA). Cylindrical implants measuring 2.5 by 2.5 mm were produced, containing approximately 0.30 mg of oxadiazole **4e** per implant. The implant was shown to be biocompatible based on cytocompatibility tests in VERO cells. Additionally, mechanical and hardness tests demonstrated that the material, even with the drug, maintains rigidity in its printed form.

Upon contact with liquids, the photopolymerized resin swells, and this behavior was also observed in the resin containing the drug. Swelling tests were conducted using PBS buffer at pH 7.4 at 25.0 and 35.7 °C. The polymer was observed to have a negative temperature response, meaning it swells less with increasing temperature. This temperature-dependent behavior was not observed in the material containing oxadiazole **4e**.

The release of oxadiazole from the polymeric matrix was studied over 50 days using PBS buffer at pH 7.4 at 25.0 and 35.7 °C. In these tests, it was observed that the release rate was very slow regardless of the temperature, not reaching 10% of the total by the end of the 50 days of testing. Extraction and immediate release studies of oxadiazole **4e** from the polymeric matrix showed that approximately 51% of the total estimated drug per implant was extracted. This indicates that, out of approximately 51% available for release, less than 10% was released in PBS after 50 days of study. Since it is believed that the release rate, in this case, is proportional to the swelling rate, and as the swelling was lower in the polymer containing the drug, the drug likely altered the density of the polymer network, making the release slower. Nevertheless, when conducting cytotoxicity tests in NCIH-292 cells with the implant containing **4e**, it was observed that the implant retained the cytotoxic activity of the isolated drug, showing potential for cancer treatment.

## Supplementary Material



## Notes

^1^H and ^13^C NMR spectroscopy and mass spectrometry data; swelling behavior tables; calibration curves for **4e** release; raw mechanical tests data; and raw data for IC_50_ value calculation (DOCX)
