# Supplementary material for: Development of Photopolymerizable Implants for Controlled Release of Pro-Apoptotic 1,2,4-Oxadiazoles
Source: ACS Omega. 2025 May 7;10(19):19314–25. doi: 10.1021/acsomega.4c09142 (PMC12096222; doi:10.1021/acsomega.4c09142)

**ACS Omega**

Supporting Information

**Development of Photopolymerizable Implants for Controlled Release of Pro-Apoptotic 1,2,4-Oxadiazoles**

Rayane C.G. Aciole, Maria J. S. Lima, Erwelly B. de Oliveira, Dayane K. D.N.Santos, Jaciana S. Aguiar, Severino Alves-Jr, Janaína V. dos Anjos

**Supporting Information for**

**Development of Photopolymerizable Implants for Controlled Release of Pro-Apoptotic 1,2,4-Oxadiazoles**

Rayane C.G. Aciole,^a^ Maria J. S. Lima,^a^ Erwelly B. de Oliveira,^b^ Dayane K. D.N.Santos,^a^ Jaciana S. Aguiar,^b^ Severino Alves-Jr^*^,^a^ Janaína V. dos Anjos^*a^

^a^Departamento de Química Fundamental, and ^b^Departamento de Antibióticos, Universidade Federal de Pernambuco, 50740-560 Recife, PE, Brazil

*severino.alvesjr@ufpe.br

*janaina.anjos@ufpe.br

**Table of contents:**

| 1. ^1^H and ^13^C NMR spectra | S-04 |
| --- | --- |
| 2. Mass spectra | S-09 |
| 3. Swelling data | S-14 |
| 4. Calibration curve data in PBS | S-15 |
| 5. Release data | S-16 |
| 6. Raw data for IC_50_ value calculation | S-17 |
| 7. Mechanical tests | S-18 |
| 8. Calibration curve data in ethanol | S-20 |

**1.^1^H and ^13^C NMR spectra of the products**

**3-(4-Methoxyphenyl)-5-phenyl-1,2,4-oxadiazole (4c):**

**Figure S1**. ^1^H-NMR spectrum of **4c** (400 MHz CDCl_3_)


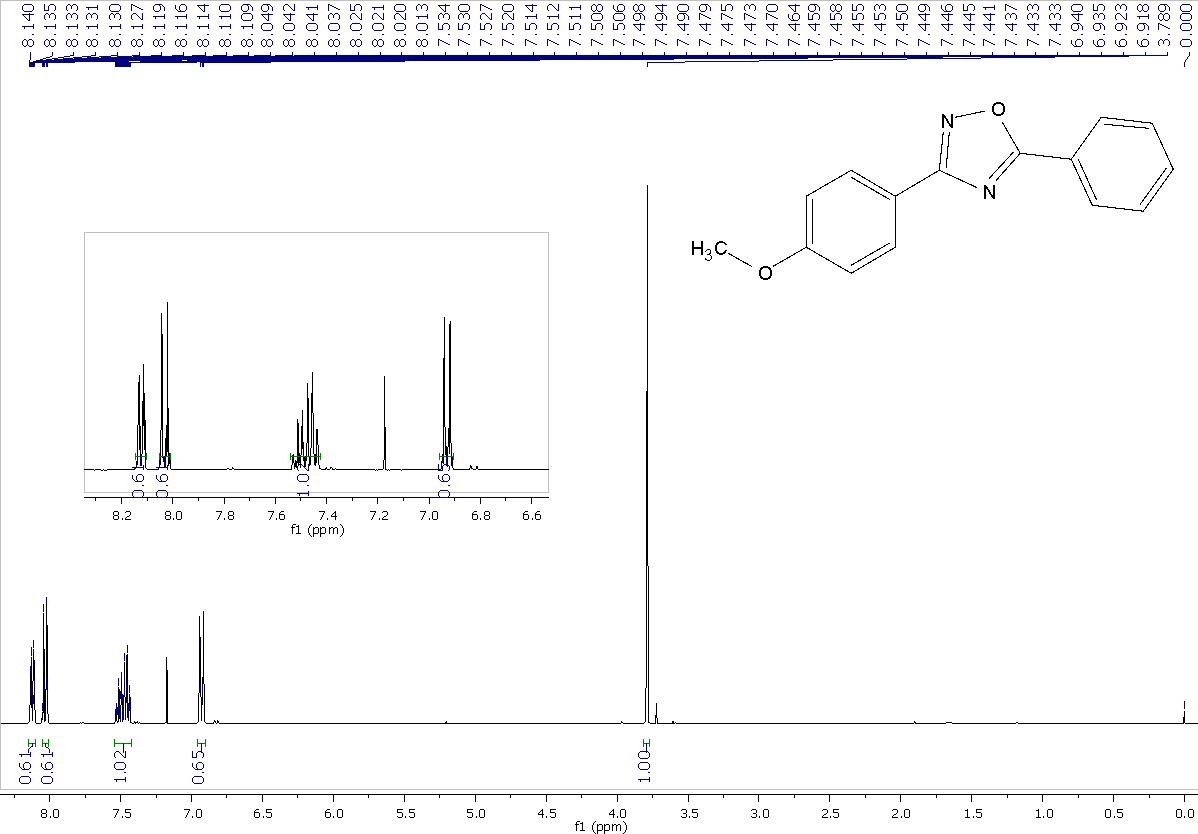


**Figura S2.** ^13^C-NMR spectrum of **4c** (100 MHz, CDCl_3_)


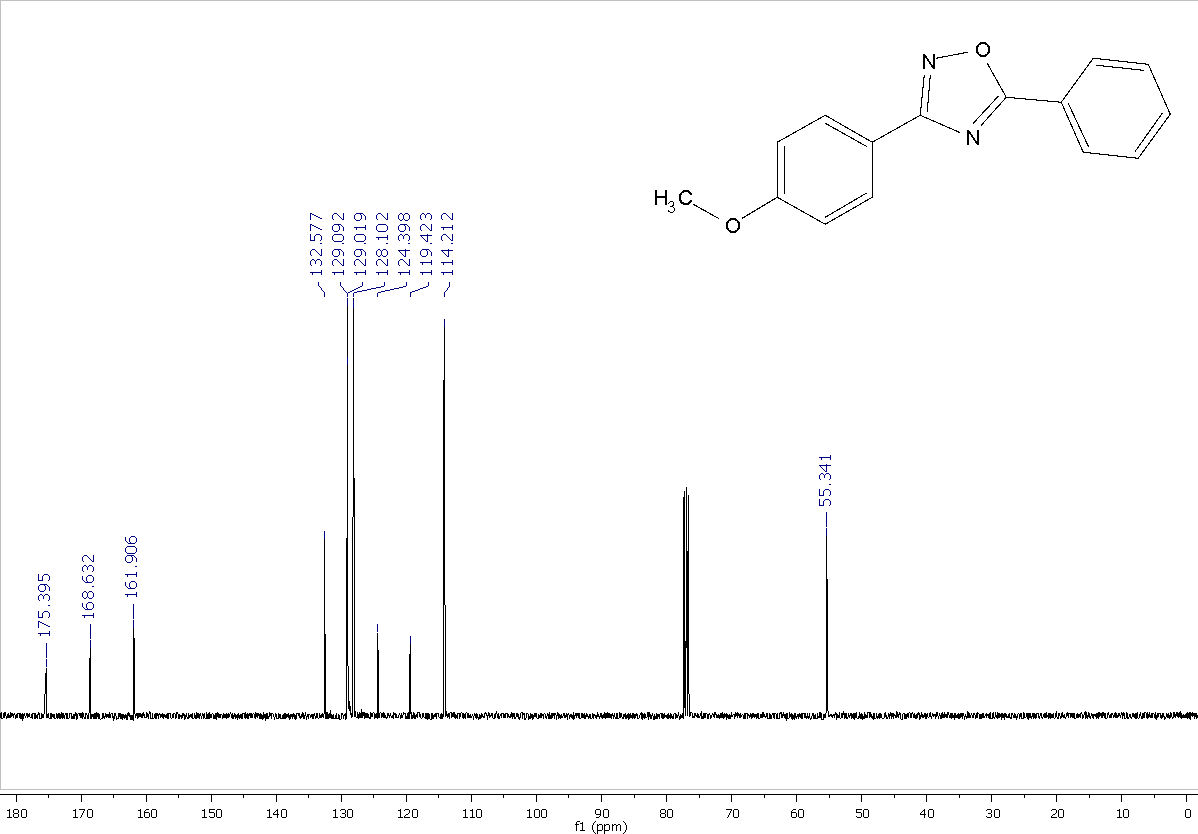


**3-(4-Chlorophenyl)-5-(furan-2-yl)-1,2,4-oxadiazole (4e):**

**Figure S3**. ^1^H-NMR spectrum of **4e** (400 MHz CDCl_3_)


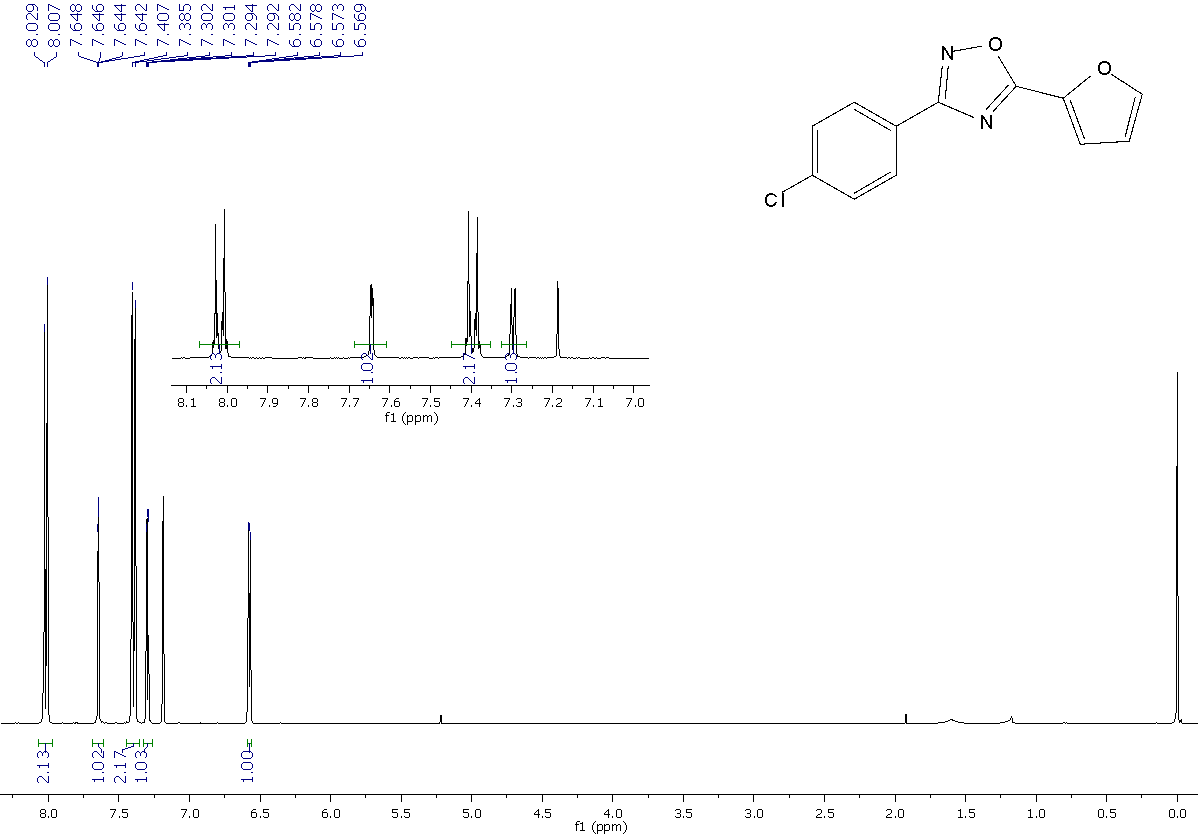


**Figure S4.** ^13^C-NMR spectrum of **4e** (100 MHz, CDCl_3_)


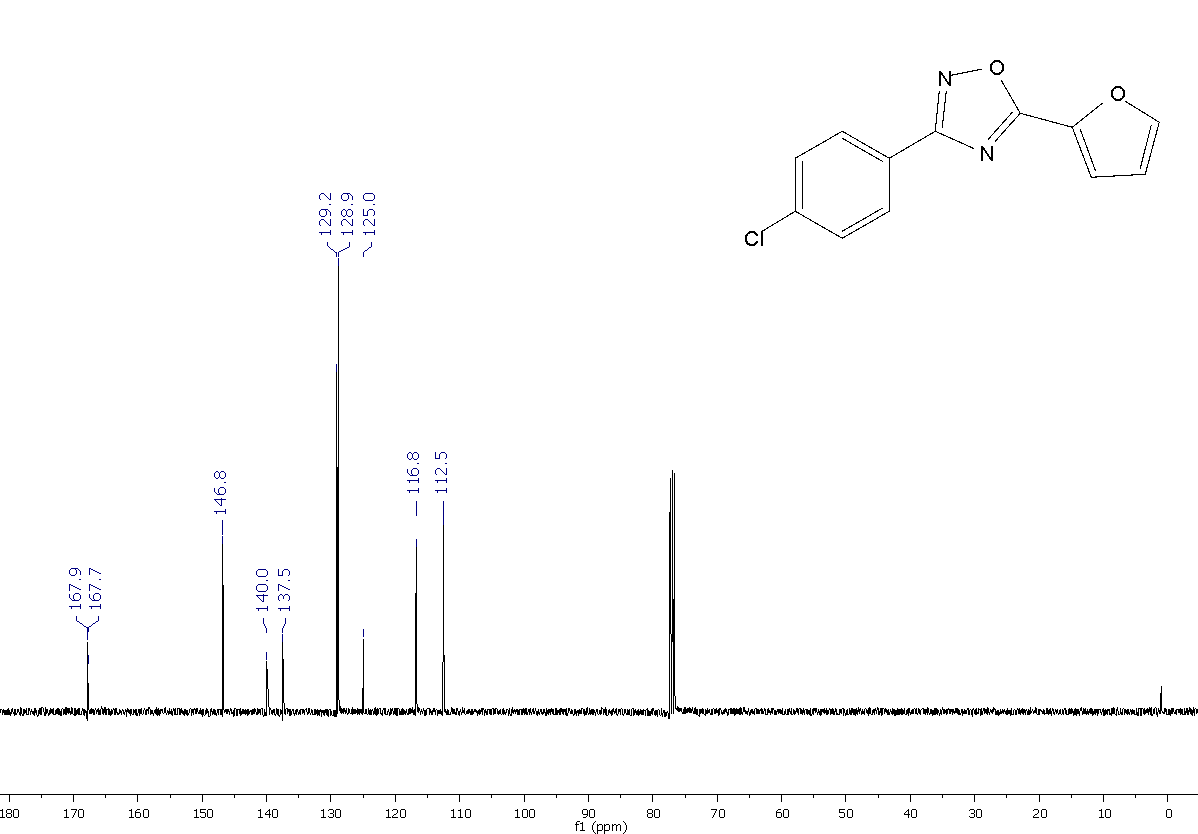


**3-(4-Methoxyphenyl)-5-(furan-2-yl)-1,2,4-oxadiazole (4f):**

**Figure S5**. ^1^H-NMR spectrum of **4f** (400 MHz CDCl_3_)


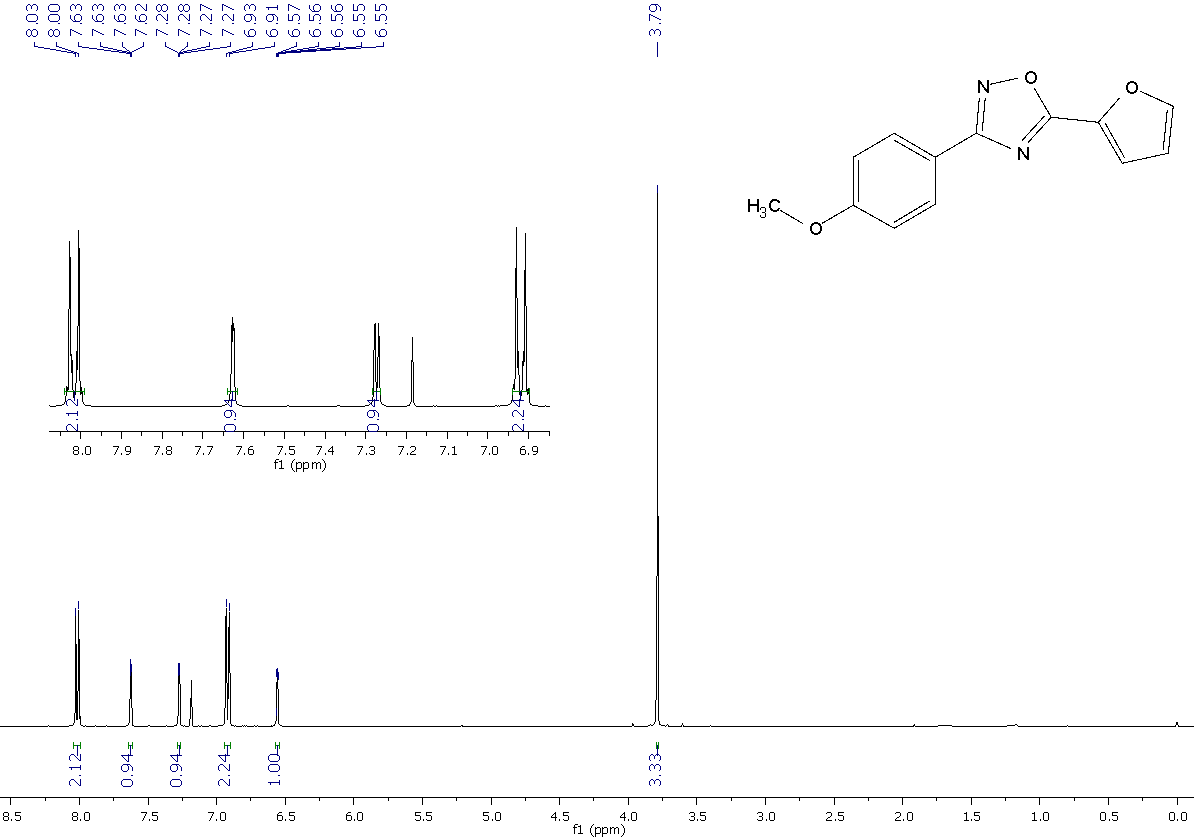


**Figure S6.** ^13^C-NMR spectrum of **4f** (100 MHz, CDCl_3_)


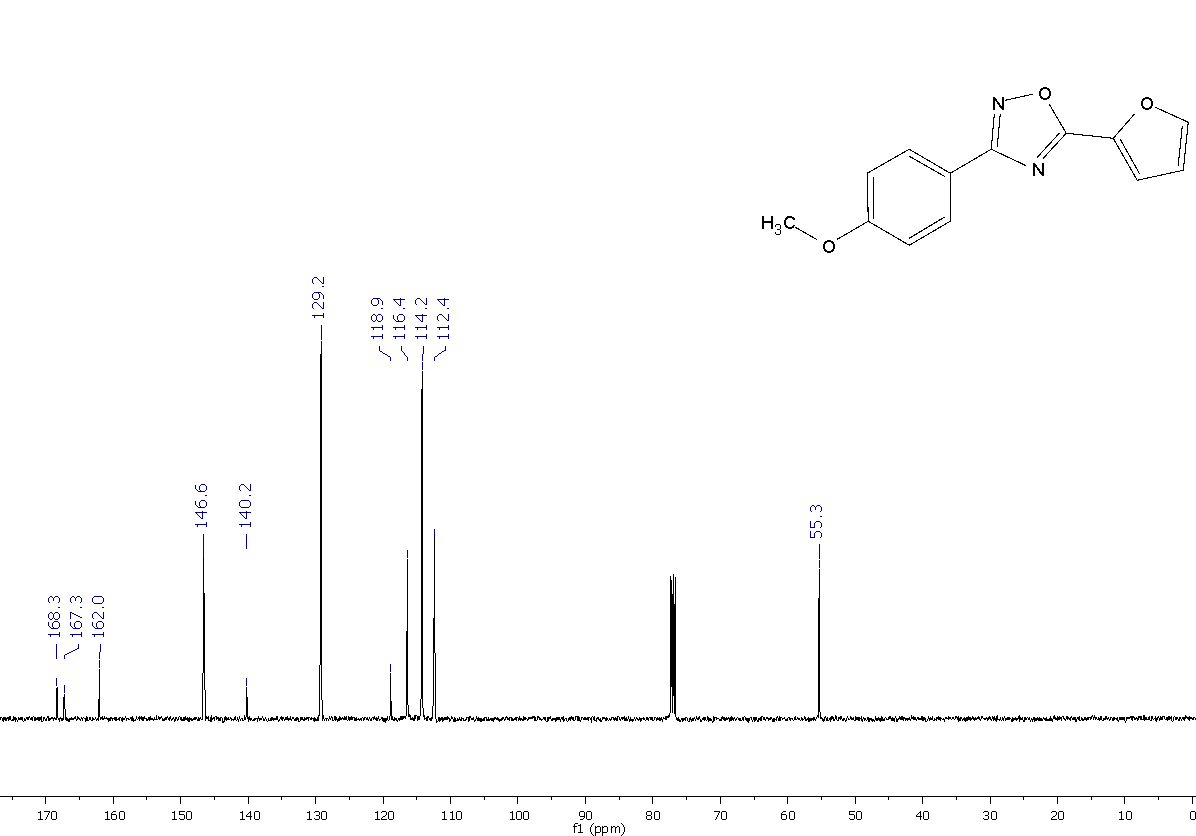


**3-(4-Chlorophenyl)-5-(thiophen-2-yl)-1,2,4-oxadiazole (4h):**

**Figure S7**. ^1^H-NMR spectrum of **4h** (400 MHz CDCl_3_)


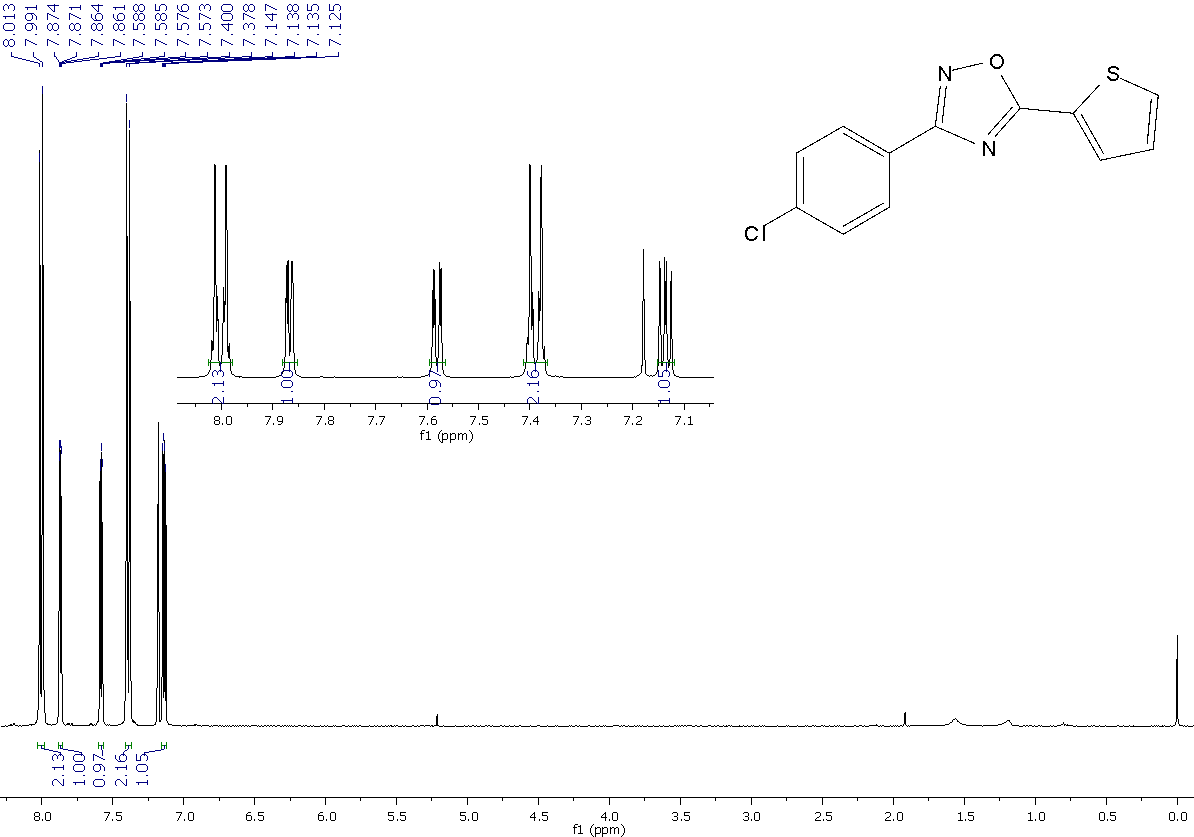


**Figure S8.** ^13^C-NMR spectrum of **4h** (100 MHz, CDCl_3_)


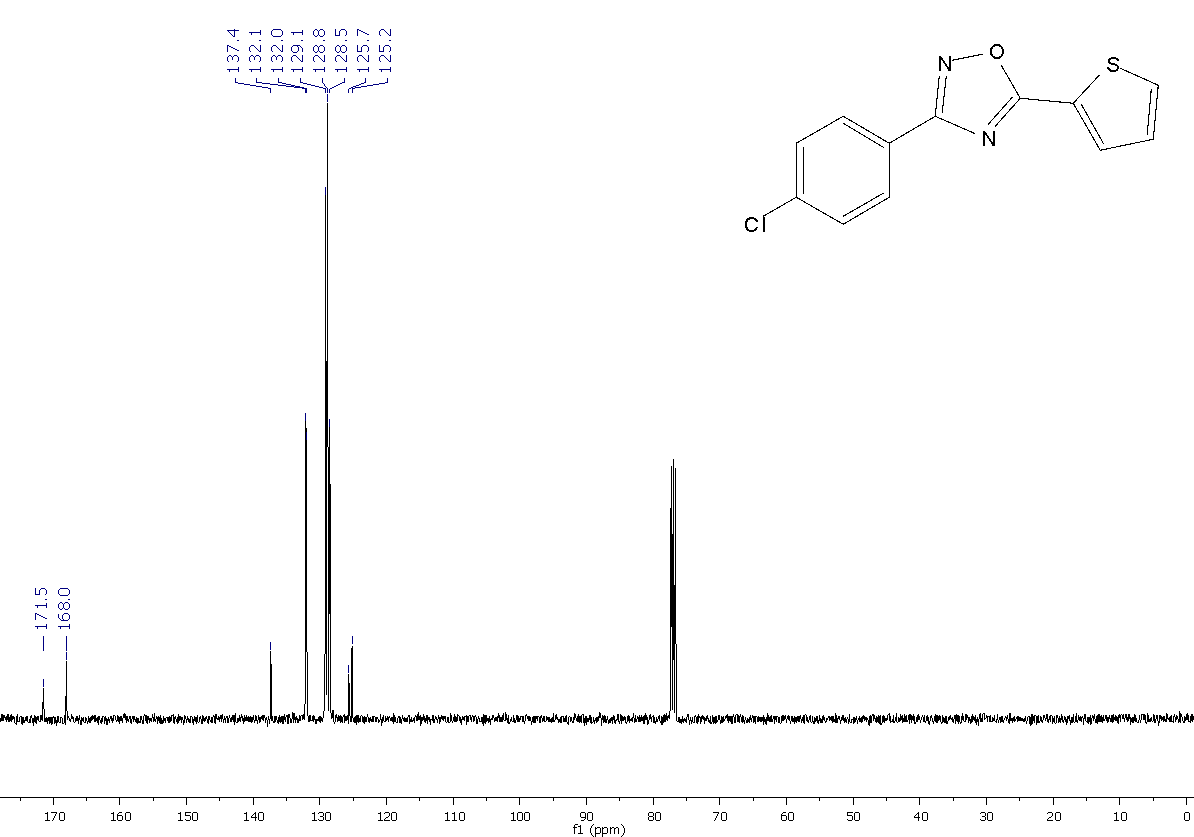


**3-(Thiophen-2-yl)-5-(furan-2-yl)-1,2,4-oxadiazole (4k):**

**Figure S9**. ^1^H-NMR spectrum of **4k** (400 MHz CDCl_3_)


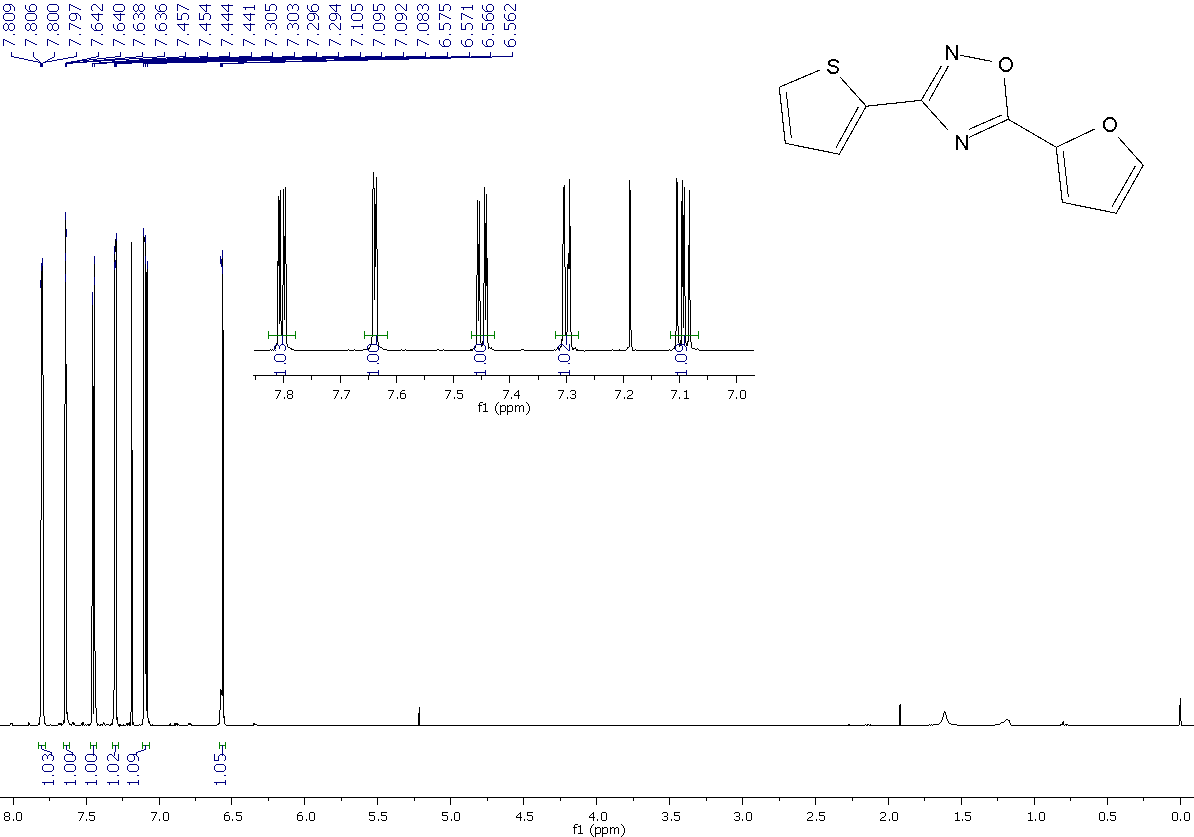


**Figure S10.** ^13^C-NMR spectrum of **4k** (100 MHz, CDCl_3_)


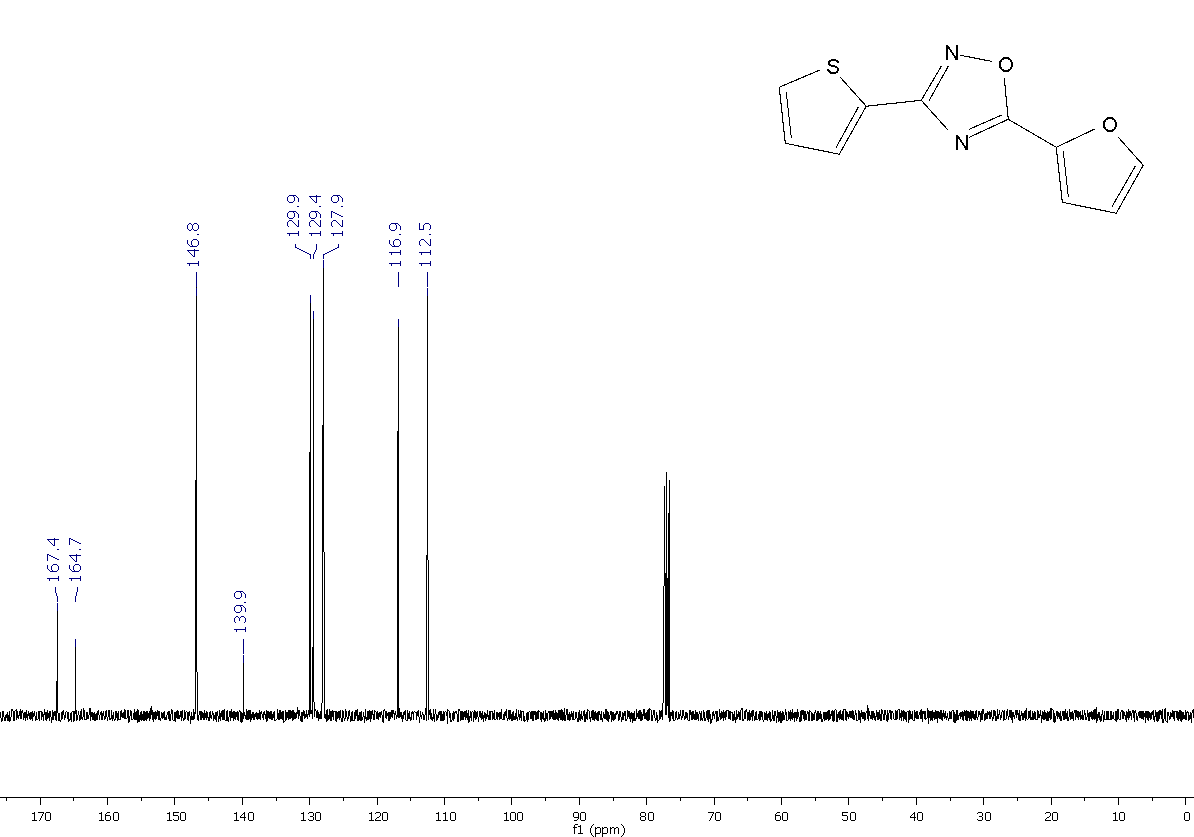


**Figure S11.** HRMS–ES of **4c** (positive mode)

**

**  ****

**Figure S12.** HRMS–ES of **4e** (positive mode)

**

**

**Figure S13.** HRMS–ES of **4f** (positive mode)

**

**

**Figure S14.** HRMS–ES of **4h** (positive mode)

**

**

**Figure S15.** HRMS–ES of **4k** (positive mode)

**

**

**Table S1:** Swelling data of implants unloaded and loaded with **4e**

| Time (h) | Unloaded polymer at 25ºC | | Loaded Polymer at 25ºC | | Unloaded polymer at 35.7ºC | | Loaded polymer at 35.7ºC | |
| --- | --- | --- | --- | --- | --- | --- | --- | --- |
|  | Polymer  mass (g) | Increase in initial mass (%) | Polymer  mass (g) | Increase in initial mass (%) | Polymer  mass (g) | Increase in initial mass (%) | Polymer  mass (g) | Increase in initial mass (%) |
| 0 | 0.0193 ± 0.0006 | - | 0.0179 ± 0.00012 | - | 0.0167 ± 0.00007 | - | 0.0162 ± 0.0001 | - |
| 24 | 0.3667 ± 0.0010 | 1900 | 0.0836 ± 0.0001 | 467 | 0.1825 ± 0.0001 | 1093 | 0.0442 ± 0.0002 | 273 |
| 48 | 0.3827 ± 0.0051 | 1983 | 0.0837 ± 0.0004 | 467 | 0.1992 ± 0.0017 | 1193 | 0.0854 ± 0.0004 | 527 |
| 72 | 0.4792 ± 0.0042 | 2483 | 0.0847 ± 0.0002 | 473 | 0.2219 ± 0.0008 | 1329 | 0.0799 ± 0.0011 | 493 |
| 96 | 0.3345 ± 0.0110 | 1733 | 0.0847 ± 0.0050 | 473 | 0.2338 ± 0.0024 | 1400 | 0.0648 ± 0.0004 | 400 |
| 120 | 0.3345 ± 0.0091 | 1733 | 0.0847 ± 0.0035 | 473 | 0.2457 ± 0.0024 | 1471 | 0.0713 ± 0.0017 | 440 |

Each point represents the mean ± S.E.M. of a triplicate.

**Table S2:** Average values of triplicates for concentration, absorbance, and error used in the construction of the calibration curve

| Average Absorbance | | Error |
| --- | --- | --- |
| Concentration | Abs |  |
| 6.25.10^-4^ | 0.16610 | 0.012665335 |
| 0.00125 | 0.28111 | 0.001218250 |
| 0.00187 | 0.37674 | 0.015045233 |
| 0.00250 | 0.52193 | 0.012766154 |
| 0.00375 | 0.83777 | 0.028161506 |
| 0.00500 | 1.04644 | 0.031926798 |

Each point represents the mean ± S.E.M. of a triplicate.

**Table S3:** Average values of triplicates for the drug release percentage over time and their respective errors

| Time (days) | Release in % (25.0 ºC) | Error | Time | Release in % (37.5 ºC) | Error |
| --- | --- | --- | --- | --- | --- |
| 0.125 | 0.81739 | 0.01453 | 0.125 | 1.74241 | 0.03649 |
| 1 | 1.56995 | 0.0018 | 1 | 2.65065 | 0.00621 |
| 2 | 2.53562 | 0.0684 | 2 | 3.15657 | 0.00512 |
| 6 | 3.40613 | 0.00233 | 6 | 4.01083 | 0.06039 |
| 9 | 4.90383 | 0.05118 | 9 | 4.57097 | 0.0723 |
| 14 | 5.75009 | 0.08998 | 14 | 4.97928 | 0.05201 |
| 21 | 6.17656 | 0.00637 | 21 | 6.02187 | 0.00848 |
| 35 | 6.63125 | 0.01949 | 35 | 6.4188 | 0.02747 |
| 42 | 7.12704 | 0.02604 | 42 | 6.57731 | 0.01699 |
| 50 | 7.42523 | 0.02817 | 50 | 6.6613 | 0.01519 |

Each point represents the mean ± S.E.M. of a triplicate.

**Table S4:** Raw data for IC_50_ value calculation

|  | HeLa | | | MCF-7 | | | NCIH-292 | | |
| --- | --- | --- | --- | --- | --- | --- | --- | --- | --- |
| Concentration (μM) | % Viability | | | % Viability | | | % Viability | | |
| 50 | 0.101 | 0.1 | 0.087 | 0.144 | 0.145 | 0.146 | 17.42466 | 14.08219 | 17.0137 |
| 25 | 0.323 | 0.338 | 0.412 | 0.369 | 0.415 | 0.393 | 30.19178 | 34.24658 | 30.10959 |
| 12.5 | 0.484 | 0.474 | 0.486 | 0.443 | 0.475 | 0.559 | 58.87671 | 59.06849 | 58.57534 |
| 6.25 | 0.491 | 0.461 | 0.477 | 0.452 | 0.507 | 0.315 | 95.9726 | 106.3014 | 93.34247 |
| 3.125 | 0.519 | 0.533 | 0.51 | 0.539 | 0.372 | 0.362 | 112.9863 | 124.6575 | 119.8356 |
| 1.5625 | 0.535 | 0.487 | 0.49 | 0.453 | 0.365 | 0.275 | 127.7534 | 132.0274 | 128.0000 |

**Figure S16.** Raw data from mechanical tests of the unloaded printed objects

**
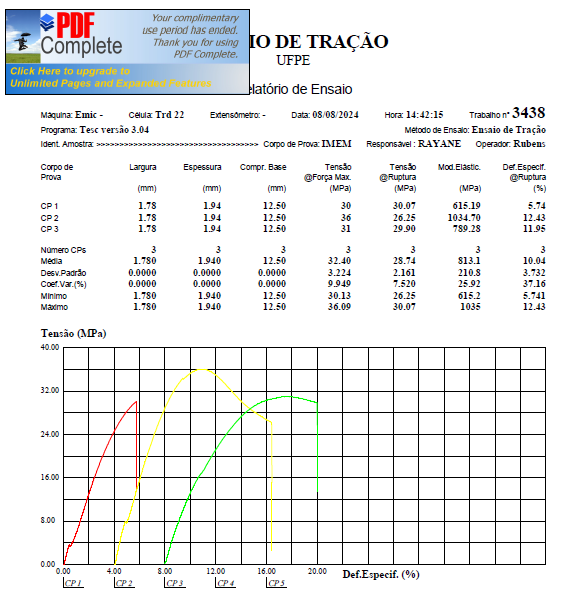
**

**Figure S17.** Raw data from mechanical tests of the drug-loaded printed objects.

**
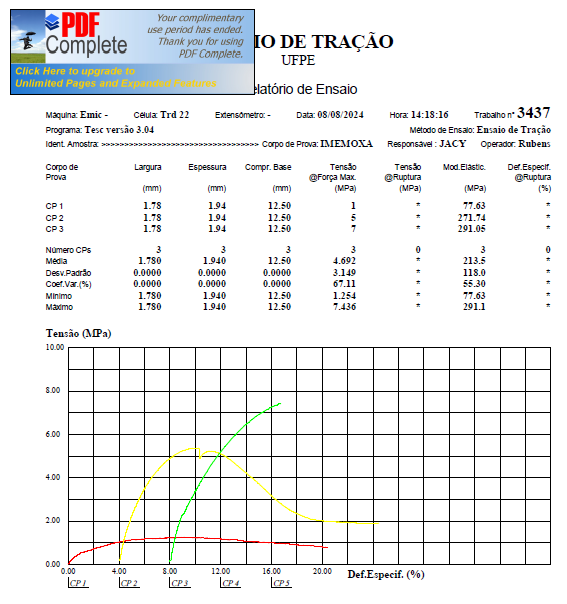
**

**Figure S18.** Calibration curve of **4e** dissolved in ethanol


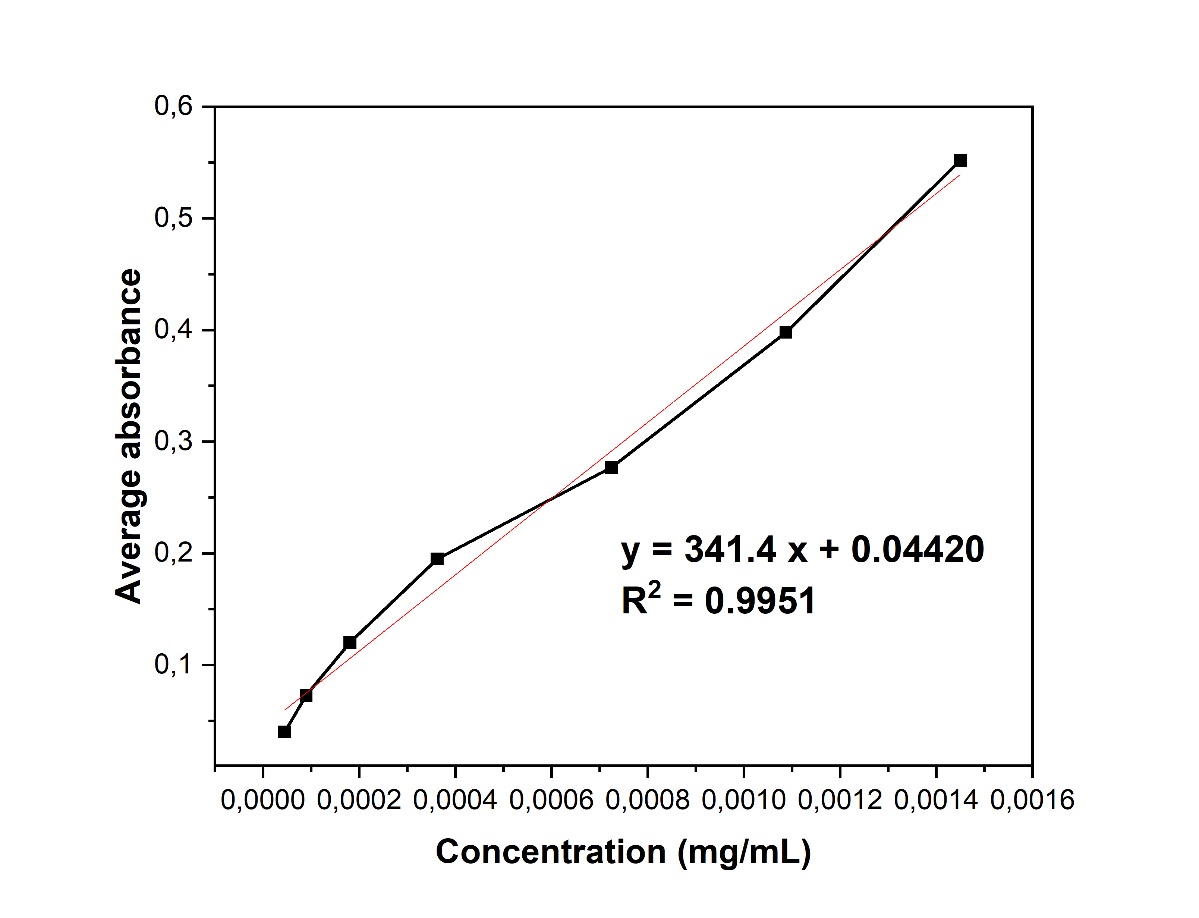


**Table S5:** Absorbance and concentration values for **4e** extraction in ethanol


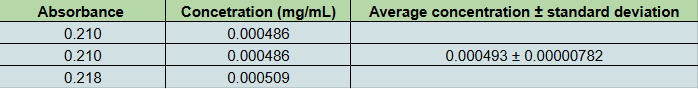

Supplement: Supplementary file 1 [file ao4c09142_si_001.docx]
